# Fasting-induced FGF21 signaling activates hepatic autophagy and lipid degradation via JMJD3 histone demethylase

**DOI:** 10.1038/s41467-020-14384-z

**Published:** 2020-02-10

**Authors:** Sangwon Byun, Sunmi Seok, Young-Chae Kim, Yang Zhang, Peter Yau, Naoki Iwamori, H. Eric Xu, Jian Ma, Byron Kemper, Jongsook Kim Kemper

**Affiliations:** 1 Department of Molecular and Integrative Physiology, University of Illinois at Urbana-Champaign, Urbana, IL 61801 USA; 2 Department of Computational Biology, School of Computer Science, Carnegie Mellon University, Pittsburgh, PA 15213 USA; 3 Proteomics Center, University of Illinois at Urbana-Champaign, Urbana, IL 61801 USA; 4 Graduate School of Bioresource and Bioenvironmental Sciences, Kyushu University, Fukuoka, 812-85812 Japan; 5 Laboratory of Structure Sciences, Van Andel Research Institute, Grand Rapids, MI 49503 USA; 6 Present Address: Korea Research Institute of Bioscience and Biotechnology (KRIBB), Gwahak-ro, Yuseong-gu, Daejeon South Korea

**Keywords:** Autophagy, Epigenetics

## Abstract

Autophagy is essential for cellular survival and energy homeostasis under nutrient deprivation. Despite the emerging importance of nuclear events in autophagy regulation, epigenetic control of autophagy gene transcription remains unclear. Here, we report fasting-induced Fibroblast Growth Factor-21 (FGF21) signaling activates hepatic autophagy and lipid degradation via Jumonji-D3 (JMJD3/KDM6B) histone demethylase. Upon FGF21 signaling, JMJD3 epigenetically upregulates global autophagy-network genes, including *Tfeb*, *Atg7*, *Atgl*, and *Fgf21*, through demethylation of histone H3K27-me3, resulting in autophagy-mediated lipid degradation. Mechanistically, phosphorylation of JMJD3 at Thr-1044 by FGF21 signal-activated PKA increases its nuclear localization and interaction with the nuclear receptor PPARα to transcriptionally activate autophagy. Administration of FGF21 in obese mice improves defective autophagy and hepatosteatosis in a JMJD3-dependent manner. Remarkably, in non-alcoholic fatty liver disease patients, hepatic expression of JMJD3, ATG7, LC3, and ULK1 is substantially decreased. These findings demonstrate that FGF21-JMJD3 signaling epigenetically links nutrient deprivation with hepatic autophagy and lipid degradation in mammals.

## Abstract (web summary)

Fasting induces hepatic autophagy to preserve energy homeostasis. Here the authors report that fasting induced fibroblast growth factor 21 signalling induces autophagy by activating lysine-specific demethylase 6B, leading to histone demethylation mediated activation of autophagy genes.

## Introduction

Lysosome-mediated autophagy is a highly conserved catabolic process that recycles cytoplasmic components, including damaged organelles and proteins, for cellular survival and maintenance of energy homeostasis under nutrient-deprived conditions^1,2^. Autophagy-mediated degradation of intracellular lipid stores, lipophagy, also plays a critical role in maintaining energy balance during nutrient deficiency by providing free fatty acids for mitochondrial fatty acid β-oxidation and ATP production^3^. Autophagy must be tightly regulated since defective autophagy has been implicated in many diseases, including cancer, neurodegenerative disease, and metabolic disorders like obesity, diabetes, and non-alcoholic fatty liver disease (NAFLD), while excessive autophagy is also harmful because it promotes cell death^4–6^.

It has long been accepted that autophagy is activated under extremely stressful conditions, but increasing evidence demonstrates that it is also regulated during feeding/fasting cycles under physiological conditions^7–10^. Nutrient-sensing factors, such as feeding-sensing FXR and SHP and fasting-sensing TFEB, CREB, and PPARα, dynamically decrease or increase, respectively, autophagic flux by modulating transcription of autophagic gene networks^7–10^. Despite the emerging importance of nuclear events in sustaining autophagy regulation, there have been only a few studies on epigenetic control in response to environmental cues. Histone acetyltransferase hMOF has a role in determining whether autophagy induction leads to cellular survival or death^11^, and the AMPK-SKP2-CARM1 signaling axis epigenetically activates transcription of autophagy-related genes after nutrient deprivation^12^. Further, histone demethylase LSD1, together with SHP, epigenetically inhibits hepatic autophagy-network genes in response to a late fed-state gut hormone, FGF19^10^. However, in vivo epigenetic regulators that convert fasting signal into induction of autophagy genes in animals are largely unknown.

Jumonji D3 (JMJD3/KDM6b) is a JmjC domain-containing histone lysine demethylase that, together with demethylases UTX and UTY, belongs to the KDM6 family and epigenetically activates genes by demethylating histone H3K27-me3^13^. JMJD3 has known functions in development, differentiation, and immunity^13^, and in extending lifespan in response to mild mitochondrial stress^14^. Recently, JMJD3 was also shown to have a metabolic function in mediating hepatic fasting responses by acting as a gene-specific transcriptional partner of SIRT1, a key cellular energy sensor^15^. JMJD3 activates transcription of direct SIRT1 target genes that promote mitochondrial fatty acid β-oxidation, including *Cpt1, Pgc-1α, and Fgf21*, but not direct SIRT1 target gluconeogenic genes^15^. Although fasting-induced JMJD3 promotes fatty acid β-oxidation, a role for JMJD3 in promoting autophagy has not been shown.

In this study, we identify a function of JMJD3 in linking nutrient deprivation to histone modifications and transcriptional induction of hepatic autophagy in mice. Under nutrient deprivation, JMJD3 is activated by the fasting-induced hepatokine, Fibroblast Growth Factor-21 (FGF21), and epigenetically upregulates global autophagy-network genes. Mechanistically, FGF21-activated PKA mediates phosphorylation of JMJD3, which is important for its nuclear localization and interaction with PPARα to transcriptionally activate autophagy. We further show that FGF21-mediated autophagy induction and lowering lipids in obese mice are dependent on JMJD3 and that the hepatic FGF21-JMJD3-autophagy axis is likely dysregulated in NAFLD patients.

## Results

### JMJD3 epigenetically activates liver autophagy gene networks

To explore epigenetic regulation of hepatic autophagy, we first examined whether selected histone modifications were altered by fasting in mouse liver. Levels of histone H3K27-me3 were markedly decreased in fasted mice, and levels of JMJD3, which activates genes by demethylating H3K27-me3^13^, were increased after fasting, whereas expression of UTX H3K27 demethylase and EZH2 H3K27 methyltransferase was unchanged (Supplementary Fig. 1). These results suggest that JMJD3 may epigenetically activate hepatic autophagy in response to nutrient deficiency.

**Fig. 1:**
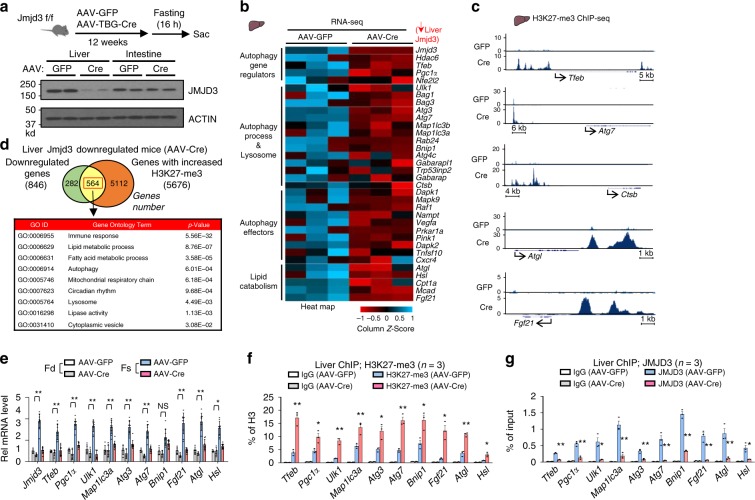
JMJD3 epigenetically activates hepatic autophagy-network genes. JMJD3-floxed mice were infected with AAV-TBG-Cre or control AAV-GFP for 12 weeks, and then, fasted for 16 h. **a** Experimental outline (top) and the levels of JMJD3 detected by IB in the liver and intestine (bottom). **b** RNA-seq: heat maps of changes in mRNA levels of autophagy-related genes (*n* = 3 mice). **c** ChIP-seq: normalized H3K27-me3 peaks at hepatic genes involved in autophagic pathways (UCSC genome browser). **d** Venn diagram (top) of downregulated genes and genes with increased H3K27-me3 levels after liver-specific downregulation of JMJD3 and G/O analysis (bottom) of the hepatic genes that show both increased H3K27-me3 levels and decreased expression. **e** The mRNA levels of the indicated genes in liver of mice fasted for 16 h (Fs) or refed (Fd) for 6 h after fasted for 10 h were measured by q-RTPCR (*n* = 5–10 mice). **f**, **g** ChIP assays: effects of the downregulation of JMJD3 on **f** H3K27-me3 levels and **g** occupancy of JMJD3 at the indicated genes (*n* = 3 mice). Source data are provided as a Source Data file. All values are presented as mean ± SD. Statistical significance was measured using the **e**–**g** two-way ANOVA with the Bonferroni post-test. *P < 0.05, **P < 0.01, and NS statistically not significant.

To examine the role of JMJD3 in global regulation of hepatic genes, including autophagy genes, JMJD3 was downregulated specifically in the liver by infection of JMJD3-floxed mice with hepatocyte-targeting AAV-TBG-Cre^15,16^ (Fig. 1a), and effects of the downregulation on global gene expression and H3K27-me3 levels were examined by RNA-seq and ChIP-seq, respectively. Expression of 846 genes was decreased by downregulation of JMJD3 (Fig. 1b, Supplementary Fig. 2a), which include genes involved in autophagy, *Ulk1, Atg3, Atg7, and Lc3;* a transcriptional activator of autophagy, *Tfeb*^7^; a lipase important for lipophagy, *Atgl*^17^; and a fasting-induced hepatokine promoting lipid catabolism, *Fgf21*^18,19^, and H3K27-me3 levels detected by ChIP-seq at nearly all of these genes were increased (Fig. 1c, Supplementary Fig. 2c). Remarkably, gene ontology (GO) analysis of 564 potential JMJD3 target genes with both decreased expression and increased levels of H3K27-me3 after downregulation of JMJD3, revealed that autophagy and lysosomal function are potentially regulated by JMJD3 in fasted mice (Fig. 1d, Supplementary Fig. 2b–d). Analysis of mRNA and H3K27-me3 levels for selected autophagy genes validated these genomic results (Fig. 1e, f) and occupancy of JMJD3 at these genes in fasted mice was decreased as expected by liver-specific downregulation of JMJD3 (Fig. 1g). These global analyses reveal a potential role for JMJD3 in epigenetic induction of hepatic autophagy.

### JMJD3 activates hepatic autophagy under nutrient deficiency

**Fig. 2:**
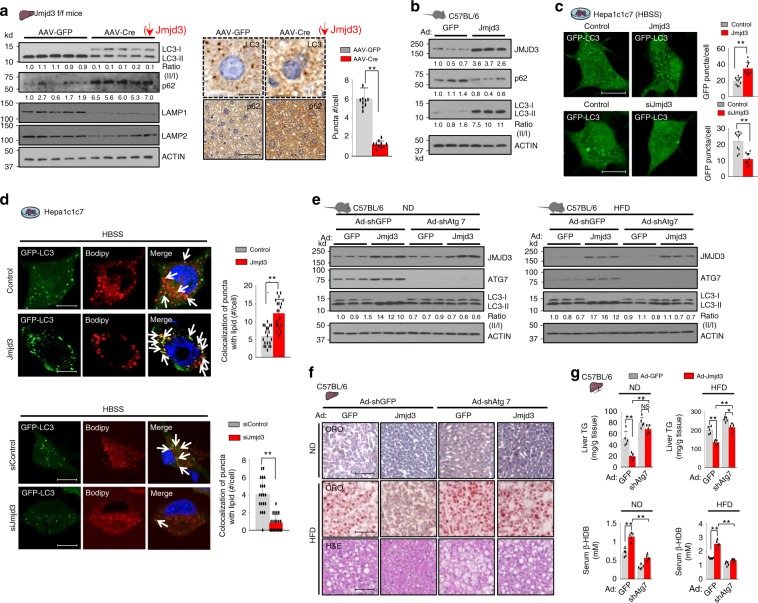
JMJD3 promotes hepatic autophagy, including lipophagy. **a** The indicated hepatic proteins were detected by IB in JMJD3-floxed mice infected with AAV-TBG-Cre or AAV-GFP for 12 weeks and fasted for 16 h. Relative band intensities for p62 and the LC3-II/LC3-I ratios are below the blots (left, *n* = 5 mice). LC3 and p62 in representative images of liver sections detected by IHC (middle) and numbers of LC3 puncta/cell (right, *n* = 10 hepatocytes) (scale bar = 10 μm for LC3, 50 μm for p62). **b** Levels of the indicated hepatic proteins were determined by IB in C57BL/6 mice infected with Ad-empty (GFP) or Ad-JMJD3 for 4 weeks and fasted for 8 h (*n* = 3 mice). **c**, **d** Hepa1c1c7 cells were transfected with expression plasmids or JMJD3 siRNA as indicated for 72 h and cultured in HBSS for 2 h. **c** Representative confocal images and the average number of GFP-LC3-II puncta/cell (right, *n* = 10 cells) are shown (scale bar = 5 μm). **d** Cells were stained for lipid droplets with BODIPY (red) and imaged by confocal microscopy. Co-localization of GFP-LC3 puncta and BODIPY in the merged image is indicated by white arrows. The number of fluorescent puncta that co-localized with lipid staining (right, *n* = 20 cells) are shown (scale bar = 5 μm). **e**–**g** C57BL/6 mice were fed a normal chow diet (ND) or high-fat diet (HFD) for 8 weeks and then were injected with adenoviruses as indicated for 4 weeks. **e** Hepatic levels of the indicated proteins were measured by IB (*n* = 3 mice). The ratios of band intensities for the LC3-I/LC3-II relative to those in the first lane are shown below the blot. **f** Lipids in liver sections stained with Oil Red O (ORO) and H&E (scale bar = 50 μm). **g** Levels of hepatic triglycerides (TG) and serum β-hydroxybutyrate (β-HDB) (*n* = 5 mice). Source data are provided as a Source Data file. All values are presented as mean ± SD. Statistical significance was measured using the (**a**, **c**, **d**) Mann–Whitney test or **g** two-way ANOVA with the Bonferroni post-test. *P < 0.05, **P < 0.01, and NS statistically not significant.

To examine whether induction of autophagy-network genes by JMJD3 actually leads to autophagy, we examined the effects of liver-specific downregulation or overexpression of JMJD3 on autophagic markers, the ratio of lipidated LC3-II to non-lipidated LC3-I, the levels of the autophagosome adapter p62, and lysosome-associated membrane protein (Lamp)1 and Lamp2^20^. The ratio of hepatic LC3-II/I, the number of LC3 puncta, and Lamp1 and 2 levels were decreased, and p62 levels were increased by downregulation of JMJD3 in mice (Fig. 2a), indicative of decreased autophagy^20^. Conversely, adenoviral-mediated liver-specific expression of JMJD3 in mice resulted in the opposite effects (Fig. 2b). Similar effects of overexpression or downregulation of JMJD3 on the number of GFP-LC3 puncta were observed in Hepa1c1c7 cells (Fig. 2c).

Downregulation of JMJD3 also decreased the LC3-II/I ratio and decreased the mRNA levels of genes involved in autophagy and lysosomal functions in primary mouse hepatocytes (PMH) (Supplementary Fig. 3a, b). Furthermore, while treatment with a lysosomal inhibitor, bafilomycin-A1, increased autophagy detected by increased LC3 puncta or ratios of LC3-II to LC3-I, overexpression of JMJD3 further increased autophagy while downregulation decreased it (Supplementary Fig. 3c–e). Similarly, treatment with an mTOR inhibitor increased the LC3-II/I ratio, and downregulation of JMJD3 decreased autophagy without reversing the phosphorylation of the mTOR target, pS6 (Supplementary Fig. 3f). These results suggest that JMJD3 activates autophagy independent of either lysosomal or mTOR action. Overall, these results, together with global studies (Fig. 1), demonstrate that JMJD3 transcriptionally activates hepatic autophagy under nutrient deprivation.

### JMJD3 promotes autophagy-mediated degradation of lipids

As key genes important for lipophagy, including *Atgl*, *Tfeb, Pgc-1α, and Fgf21*^7,17,21^, are direct targets of JMJD3 (Fig. 1, Supplementary Fig. 2), and JMJD3 promotes fatty acid β-oxidation^15^, we further examined whether JMJD3 activates hepatic lipophagy.

Overexpression of JMJD3 in Hepa1c1c7 cells increased the number of GFP-LC3 puncta that co-localized with BODIPY-stained lipids and the opposite effects were observed after downregulation of JMJD3 (Fig. 2d). Furthermore, in electron microscopy studies, autophagic vesicles within lipid droplets were observed in mouse liver after adenoviral-mediated expression of JMJD3 (Supplementary Fig. 4a). Exogenous expression of JMJD3 in livers of mice fed a normal chow diet (ND) increased the LC3-II/I ratio (Fig. 2e), decreased hepatic lipid (Fig. 2f, Supplementary Fig. 4b) and triglyceride (TG) levels, and increased serum ketone body levels (Fig. 2g). In mice fed a high-fat diet (HFD), expression of JMJD3 resulted in increased autophagy and decreased hepatic TG levels, but these JMJD3-mediated effects were blunted in autophagy-defective Atg7-downregulated mice (Fig. 2e–g). These results suggest that JMJD3 promotes lipophagy, which contributes to decreased liver TG levels and that autophagy is important for the JMJD3-mediated lipid-lowering effects.

### Fasting-induced autophagy is blunted in FGF21-LKO mice

Fasting increased JMJD3 occupancy and decreased histone H3K27-me3 levels at selected genes promoting lipophagy, including *Tfeb, Ulk1, Atgl*, *and Fgf21*, in mice (Supplementary Fig. 5). As the fasting-induced FGF21 promotes lipid catabolism^18,19^ and lysosomal function^21^, we examined whether FGF21 has a role in JMJD3-induced autophagy using liver-specific FGF21-knockout (FGF21-LKO) mice^22^.

**Fig. 3:**
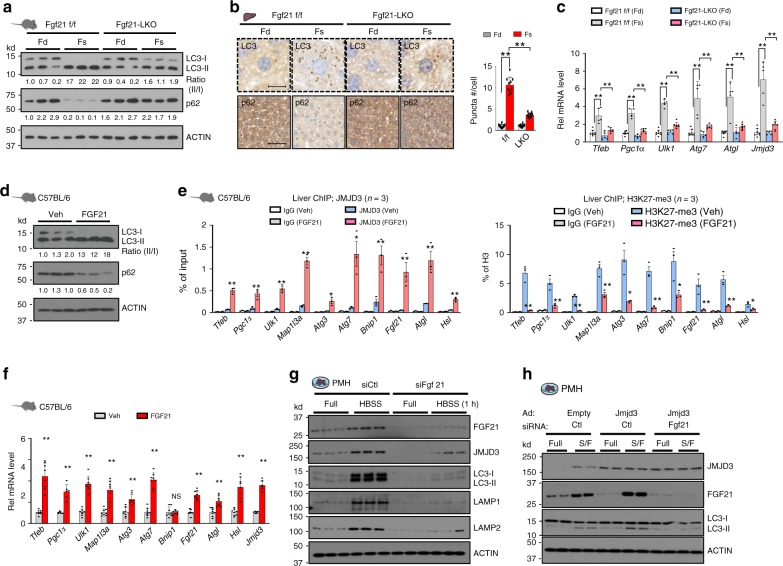
Fasting-induced FGF21 promotes hepatic autophagy. **a**–**c** FGF21 floxed or FGF21-LKO mice were fasted (Fs) for 24 h, or refed (Fd) for 24 h after fasting. **a** LC3 and p62 levels in liver extracts detected by IB. The ratios of the LC3-II/I and p62 band intensities are shown below the blot. (*n* = 3 mice). **b** LC3 or p62 was detected by IHC analysis. Representative images of liver sections and the average number of LC3-II puncta/cell (right, *n* = 10 hepatocytes) are shown (scale bar = 10 μm for LC3, 50 μm for p62). **c** The mRNA levels of the indicated genes measured by q-RTPCR (*n* = 5–8 mice). **d** The indicated hepatic proteins detected by IB with the ratios of the LC3-II/I with the band intensities of p62 relative to the first lane shown below the blot (*n* = 3 mice). **e**, **f** C57BL/6 mice were treated with FGF21 (0.1 mg/kg) for 3 h. **e** Effects of FGF21 on occupancy of JMJD3 (left) and H3K27-me3 levels (right) at the indicated genes (*n* = 3 mice). **f** Hepatic mRNA levels measured by q-RTPCR (*n* = 6–8 mice). **g** Effects of siRNA-mediated downregulation of FGF21 on levels of indicated proteins in PMH incubated with M199 or HBSS medium. **h** PMH were transfected with siFGF21 or control RNA for 48 h and infected with Ad-JMJD3 or Ad-empty for 24 h. Cells were cultured in serum-free or complete M199 medium for 12 h and levels of the indicated proteins were determined by IB (*n* = 2 culture dishes). Source data are provided as a Source Data file. All values are presented as mean ± SD. Statistical significance was measured using the **f** Mann–Whitney test or **b**, **c**, **e** two-way ANOVA with the Bonferroni post-test. *P < 0.05, **P < 0.01, and NS statistically not significant.

The increased ratio of LC3-II/I, decreased p62 levels, and increased LC3 puncta (Fig. 3a,b), and induction of *Tfeb, Pgc-1α, Ulk1, Atg7, Atgl, and JMJD3* (Fig. 3c) observed after fasting of control mice were attenuated in FGF21-LKO mice. These results suggest that physiological levels of endogenous FGF21 induced by fasting promote hepatic autophagy in an autocrine-manner. Consistent with these results, treatment with FGF21 increased JMJD3 occupancy and demethylation of H3K27-me3 at numerous autophagy genes, and increased expression of these genes (Fig. 3e, f).

In hepatocytes, the increased levels of LC3, Lamp1/2, and JMJD3 expression in nutrient-deprived medium were attenuated by downregulation of FGF21 (Fig. 3g). Moreover, expression of JMJD3 increased the LC3-II/I ratio but this JMJD3-mediated effect was also blunted by FGF21 downregulation (Fig. 3h). These findings reveal a critical role for hepatic FGF21 in hepatic autophagy induced either by nutrient deprivation or overexpression of JMJD3.

### JMJD3 has a critical role in FGF21-induced hepatic autophagy

**Fig. 4:**
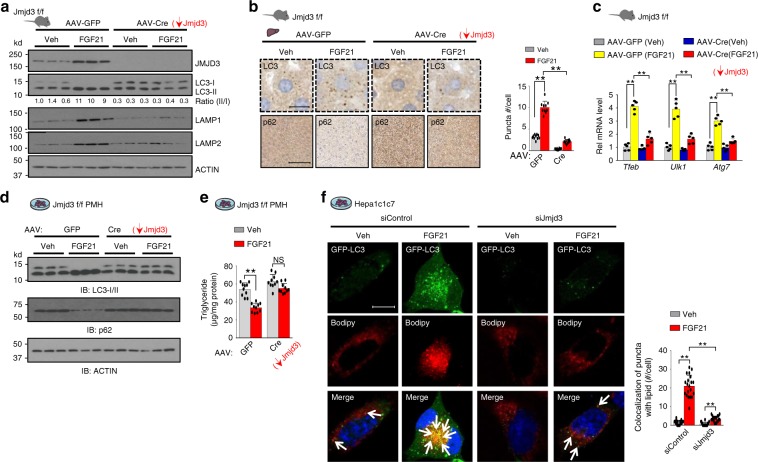
FGF21-induced hepatic autophagy is largely dependent on JMJD3. **a**–**c** JMJD3-floxed mice were infected with AAV-TBG-Cre or AAV-GFP for 12 weeks (*n* = 6 mice/group), and injected i.v. with vehicle or FGF21 (0.1 mg/kg) for 3 h. **a** The indicated hepatic proteins were detected by IB (*n* = 3 mice). **b** LC3 or p62 was detected in liver sections by IHC and the number of LC3-II puncta/cell were quantified (right, *n* = 10 hepatocytes) (scale bar = 10 μm for LC3, 50 μm for p62). **c** Hepatic mRNA levels of the indicated autophagy-related genes measured by q-RTPCR (*n* = 5 mice). **d**, **e** PMH from JMJD3-floxed mice were infected with AAV-TBG-GFP or AAV-TBG-Cre for 72 h, and treated with vehicle or FGF21 (100 ng/ml) for 12 h. **d** Levels of the indicated proteins measured by IB (*n* = 3 culture dishes). **e** Levels of cellular triglycerides (TG) (*n* = 10 culture dishes). **f** Hepa1c1c7 cells were transfected with GFP-LC3 plasmid and with control (siC) or JMJD3 siRNA as indicated. After 72 h, cells were supplemented with 400 μM oleic acid for 6 h before incubation in serum-free DMEM containing vehicle or 100 ng/ml FGF21 for 12 h. Cells were stained for lipid droplets with BODIPY (red) and imaged by confocal microscopy. Co-localization of GFP-LC3 puncta (green) and BODIPY in the merged images is indicated by white arrows. The average number/cell (right, *n* = 20 cells) of fluorescent puncta that co-localized with lipid staining is shown (scale bar = 5 μm). Source data are provided as a Source Data file. All values are presented as mean ± SD. Statistical significance was measured using the (**b**, **c**, **e**, **f**) two-way ANOVA with the Bonferroni post-test. **P < 0.01, and NS statistically not significant.

As JMJD3 epigenetically induces hepatic expression of *Fgf21* in response to fasting^15^, we further examined whether JMJD3 is also important for FGF21-induced hepatic autophagy. FGF21 treatment in mice increased the LC3II/I ratio and Lamp1/2 levels (Fig. 4a), LC3 puncta (Fig. 4b), and expression of *Tfeb, Ulk1*, and *Atg7* (Fig. 4c), but these FGF21-mediated effects on autophagy were markedly blunted by downregulation of hepatic JMJD3. Consistent with these findings in mice, in primary mouse hepatocytes (PMH), FGF21 treatment increased p-ERK levels and the LC-II/I ratio and decreased p62 levels (Supplementary Fig. 6a). Furthermore, treatment with FGF21, but not rapamycin, increased expression of JMJD3 (Supplementary Fig. 6b). These results indicate that JMJD3 is important for hepatic autophagy induced by FGF21. As JMJD3 induces FGF21 expression and FGF21 signaling activates JMJD3, which is required for FGF21 induction of autophagy, these findings reveal an intriguing feedforward loop between FGF21 and JMJD3 to activate hepatic lipophagy upon nutrient deprivation.

### FGF21 can directly act on hepatocytes and induce lipophagy

FGF21 lowers TG levels in liver, but it remains controversial whether the liver is a direct target organ for the FGF21 action or indirectly targeted through the CNS^18,19,23,24^. We, thus, examined whether FGF21-mediated autophagy observed in mice can also occur in isolated hepatocytes. FGF21 treatment increased autophagy and decreased cellular TG levels in PMH (Fig. 4d, e), and increased autophagy gene expression (Supplementary Fig. 6c). Each of these FGF21-mediated effects was blunted by downregulation of JMJD3. Furthermore, FGF21 treatment increased co-localization of LC3 puncta and lipids in Hepa1c1c7 cells, but these effects were blunted by JMJD3 downregulation (Fig. 4f). These results suggest that FGF21 can directly act on hepatocytes and induce lipophagy, which is associated with decreased TG levels.

### PPARα is a key component of the FGF21-JMJD3-autophagy axis

**Fig. 5:**
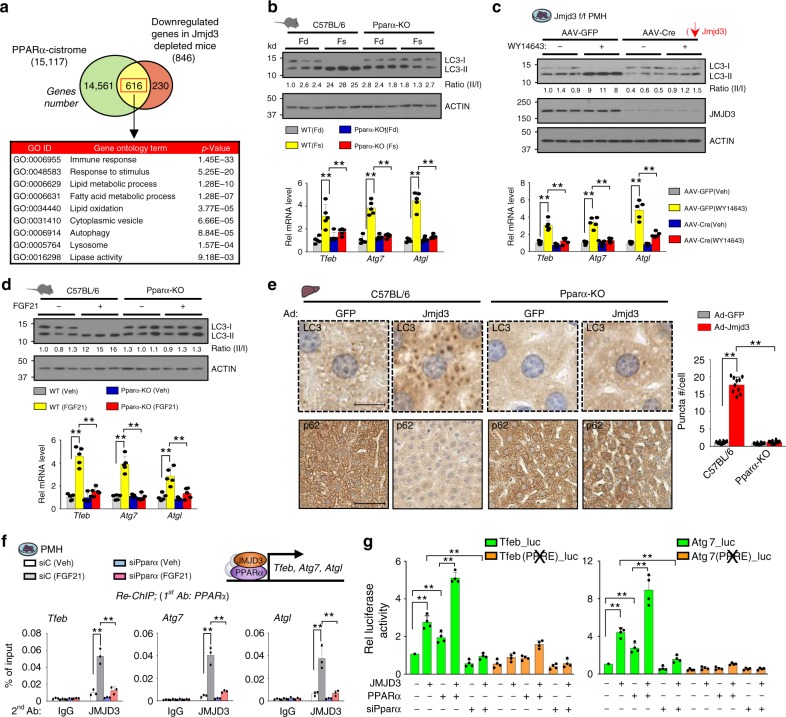
JMJD3 coactivates PPARα to induce hepatic autophagy. **a** Venn diagram (top) for genes with PPARα cistrome detected by ChIP-seq and hepatic genes inhibited by liver JMJD3 downregulation (as shown in Fig. 1b). G/O analysis (bottom) of the overlapping genes. **b** WT or PPARα-KO mice were fasted (Fs) for 24 h or refed for 24 h (Fd) after fasting. Levels of LC3 in liver extracts measured by IB with the LC3-II/I ratios shown below the blot (top, *n* = 3 mice). The mRNA levels of indicated genes (bottom, *n* = 5 mice). **c** PMH from JMJD3-floxed mice were infected with AAV-GFP or AAV-Cre for 72 h and treated with vehicle or WY14643 for 12 h. The indicated proteins were detected by IB with the LC3-II/I ratios shown below the blot (top, *n* = 3 culture dishes). The mRNA levels of indicated genes (bottom, *n* = 5 mice). **d** C57BL/6 or PPARα-KO mice were fasted for 1 h and treated with vehicle or 0.1 mg/kg FGF21 for 3 h. Hepatic levels of LC3 measured by IB with the LC3-II/I ratios shown below the blot (top, *n* = 3). The mRNA levels of indicated genes (bottom, *n* = 5 mice). **e** LC3 and p62 detected by IHC. Representative images of liver sections (left) and the average number of puncta/cell (right, *n* = 10 hepatocytes) are shown (scale bar = 10 μm for LC3, 50 μm for p62). **f** re-ChIP: Hepatocytes were transfected with PPARα siRNA, 72 h later, cells were treated with FGF21 for 2 h. PPARα was immunoprecipitated followed by immunoprecipitation of JMJD3, and enrichment of *Tfeb*, *Atg7,* and *Atgl* sequences was determined (*n* = 3 culture dishes). **g** Hepa1c1c7 cells were transfected with a luciferase reporter containing the PPARα binding site or mutated site from *Tfeb* or *Atg7* and with plasmids and siRNAs as indicated. After 36 h, the cells were treated with 50 μM WY14643 and 100 ng/ml FGF21 overnight. Luciferase activities were normalized to β-galactosidase activities (*n* = 4 culture dishes). Source data are provided as a Source Data file. **b**–**g** Values are presented as mean ± SD. Statistical significance was measured using the **g** one- or **b**–**f** two-way ANOVA with the Bonferroni post-test. **P < 0.01.

We next examined the mechanism by which JMJD3 transmits the FGF21 signal to epigenetically activate autophagy genes. We first identified transcriptional factors that might recruit JMJD3 to autophagy genes by examining candidate factors that are known to promote autophagy, PPARα^7,9^, CREB^8^, and FOXO1^12^. The increase in the LC3-II/I ratio induced by JMJD3 expression was largely blocked by downregulation of PPARα, while a substantial increase was still observed after downregulation of CREB or FOXO1 (Supplementary Fig. 7a). Comparative analyses of our RNA-seq data (Fig. 1b, Supplementary Fig. 2a) with published PPARα microarray^25^ or ChIP-seq^9^ data revealed that ~70% of hepatic genes downregulated in JMJD3-depleted mice had binding peaks for PPARα (Fig. 5a) and ~70% of the hepatic genes downregulated in PPARα-KO mice also had increased levels of histone H3K27-me3 (Supplementary Fig. 7b). These genes, potentially regulated by both PPARα and JMJD3, were involved in autophagy, lysosome, and lipid catabolism based on GO analysis (Fig. 5a).

To determine the importance of PPARα in the FGF21-JMJD3-autophagy axis, we utilized PPARα-KO mice. Fasting increased LC3-II/I ratios and autophagy gene expression in control mice, but these effects were blunted in PPARα-KO mice (Fig. 5b). Conversely, activation of PPARα by treatment with an agonist, WY14643, increased LC3-II/I ratios and expression of autophagy genes in a JMJD3-dependent manner in PMH (Fig. 5c) and increased the interaction of JMJD3 with PPARα (Supplementary Fig. 7c). The effects of FGF21 treatment (Fig. 5d) or JMJD3 expression (Fig. 5e, Supplementary Fig. 7d) on autophagy were also blunted in PPARα-KO mice. Further, FGF21 treatment increased co-occupancy of JMJD3 and PPARα at *Tfeb, Atg7*, and *Atgl* genes (Fig. 5f). Consistent with these results, the interaction of JMJD3 with PPARα was increased after treatment with FGF21, but not with rapamycin (Supplementary Fig. 7e, f). In reporter assays, expression of JMJD3 enhanced PPARα-mediated transactivation of *Tfeb-luc and Atg7-luc* (Fig. 5g). Collectively, these findings demonstrate that PPARα is a key component of the induction of autophagy network genes mediated by the fasting-triggered FGF21-JMJD3 axis.

### JMJD3 phosphorylation at T1044 is critical for its function

**Fig. 6:**
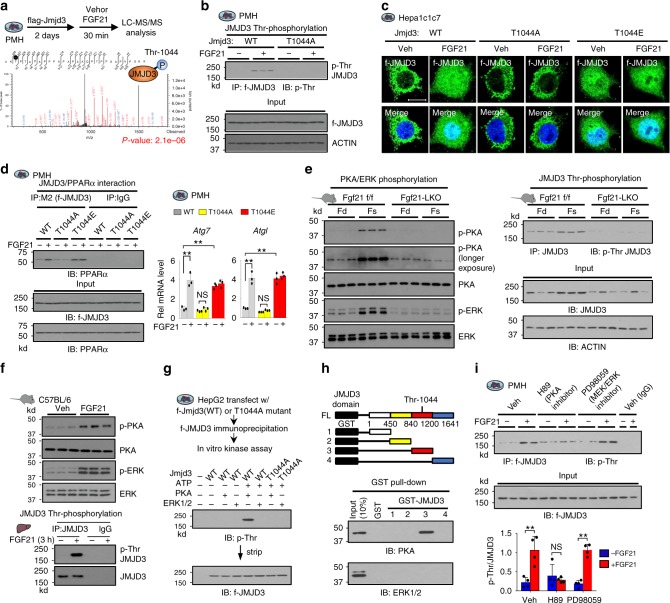
Phosphorylation of JMJD3 by FGF21-activated PKA is critical for autophagy induction. **a** Experimental outline (top) and spectrum from LC-MS/MS analysis identifying a JMJD3 peptide containing phosphorylated Thr-1044 (bottom). **b** PMH were transfected with plasmids as indicated, and after 48 h, were treated with vehicle or FGF21 (100 ng/ml) for 30 min. p-Thr-JMJD3 levels were detected by IP/IB and input protein by IB (*n* = 3 culture dishes). **c** Hepa1c1c7 cells were transfected with JMJD3 expression plasmids and treated with vehicle or FGF21 for 30 min. Flag-JMJD3 (green) was detected by immunofluorescence (scale bar = 5 μm). **d** PMH were transfected with JMJD3 expression plasmids as indicated and treated with vehicle or FGF21 for 30 min for CoIP (left) or 2 h for qRT-PCR (right). PPARα in flag-JMJD3 immunoprecipitates or in input detected by IB (left) and the mRNA levels of the indicated genes (right, *n* = 3 culture dishes). **e** FGF21-floxed or -LKO mice were fasted for 24 h or refed for 24 h after fasting. Levels of proteins were detected by IB (left) and p-Thr JMJD3 were detected by IP/IB (right) (*n* = 3 mice). **f** C57BL/6 mice were treated with 0.1 mg/kg FGF21 for 3 h and then, phosphorylated levels of PKA, ERK, and JMJD3 were detected by IB (*n* = 3 mice). **g** Immunoprecipitated flag-JMJD3 was incubated with ATP, PKA, or ERK1/2 as indicated and levels of p-Thr-JMJD3 were detected by IB. **h** Schematic of fragments of JMJD3 that were fused to GST (top). Binding of PKA or ERK1/2 to GST-JMJD3 proteins was detected by IB (bottom). **i** PMH were treated with FGF21 and with vehicle or a PKA (H89,10 μM) or MEK/ERK (PD98059, 40 μM) inhibitor for 30 min. Levels of p-Thr-JMJD3 were detected by IP/IB (top) and quantified (bottom, *n* = 4 culture dishes). Source data are provided as a Source Data file. **d**, **i** Values are presented as mean ± SD. Statistical significance was measured using (**d**, **i**) two-way ANOVA with the Bonferroni post-test. **P < 0.01, and NS statistically not significant.

We further investigated the mechanism by which FGF21 signaling activates JMJD3. In mass spectrometry analysis, Thr-1044 was the only phosphorylation site detected in JMJD3 from FGF21-treated hepatocytes (Fig. 6a, Supplementary Fig. 8a). Indeed, p-Thr JMJD3 levels for WT-JMJD3, but not T1044A-JMJD3, were increased by FGF21 treatment in PMH (Fig. 6b, Supplementary Fig. 8b). These results indicate that JMJD3 is phosphorylated at Thr-1044 in response to FGF21.

We next examined the role of the FGF21 signal-induced JMJD3 phosphorylation in induction of autophagy genes. JMJD3 was detected in the cytoplasmic and mitochondrial fractions and FGF21 treatment increased nuclear localization of JMJD3 in PMH (Supplementary Fig. 8c, d) and also in Hepa1c1c7 cells (Fig. 6c). Further, FGF21 treatment increased the interaction of JMJD3 with PPARα, the expression of *Atgl, Atg7*, and *Tfeb* and the LC3 II/I ratio (Fig. 6d, Supplementary Fig. 8e, f). These FGF21-mediated effects were blocked by the p-defective T1044A mutation of JMJD3, while effects similar to those in FGF21-treated cells were observed even in vehicle-treated cells with a p-mimic T1044E mutation (Fig. 6c, d, Supplementary Fig. 8d–f). Notably, FGF21-induced phosphorylation of JMJD3 was detected in both the cytoplasm and nucleus of FGF21-treated cells, while the interaction of JMJD3 with PPARα was detected only in the nucleus (Supplementary Fig. 8g). Collectively, these results indicate that FGF21-induced phosphorylation of JMJD3 is critical for its activation and for the induction of autophagy genes.

### FGF21-activated PKA mediates the phosphorylation of JMJD3

Analysis of JMJD3 sequence adjacent of Thr-1044 revealed motifs for several kinases, including PKA, as well as, a well-known FGF21 signaling kinase, ERK^18,19^ (Supplementary Fig. 9a). Fasting overnight increased the phosphorylation of JMJD3 and both PKA and ERK in control mice, but not in FGF21-LKO mice (Fig. 6e), consistent with mediation of the phosphorylation by fasting-induced FGF21. Indeed, FGF21 treatment in mice increased phosphorylation of JMJD3 and both PKA and ERK (Fig. 6f) and increased the interaction of JMJD3 with PKA, but not with ERK (Supplementary Fig. 9b). In in vitro kinase assays, PKA, but not ERK, phosphorylated WT-JMJD3, but not T1044A-JMJD3 (Fig. 6g) and in GST pull down assays, PKA directly interacted with JMJD3 through the domain containing Thr-1044 (Fig. 6h, Supplementary Fig. 9c). Importantly, in PMH, FGF21-induced phosphorylation of JMJD3 was significantly blunted by treatment with an inhibitor of PKA, but not of MEK/ERK (Fig. 6i). These findings indicate that FGF21 signal-activated PKA mediates the phosphorylation of JMJD3, which is important for induction of autophagy.

### Beneficial FGF21 effects on fatty liver are JMJD3-dependent

**Fig. 7:**
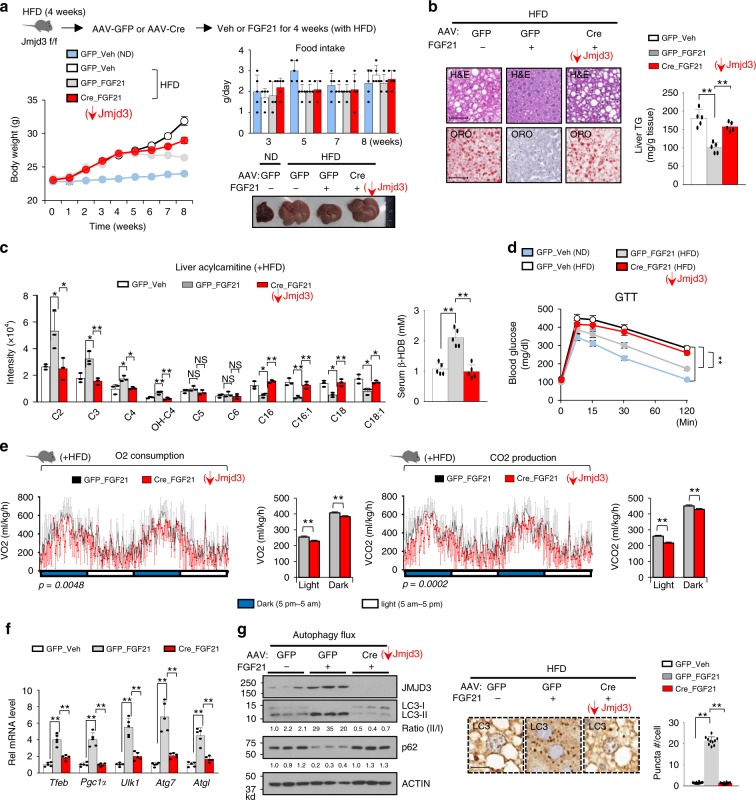
FGF21-mediated reversal of defective autophagy and hepatosteatosis in obese mice is dependent on JMJD3. JMJD3-floxed mice fed a HFD for 4 weeks were injected with the indicated viruses and then treated with vehicle or FGF21 every 2 days for 4 weeks with continued feeding of a HFD. **a**–**f** *n* = 5 mice/group, **g** *n* = 3 mice/group. **a** Experimental outline (top), body weights, food intakes, and images of liver. **b** Neutral lipids in liver sections stained with H&E and Oil Red O detected by IHC (left) and liver TG levels (right) (scale bar = 50 μm). **c** Levels of liver acylcarnitine species (left) and serum β-hydroxybutyric acid (β-HDB) (right). **d** Glucose tolerance test (GTT). **e** O_2_ consumption (left) and CO_2_ production (right) rates measured by indirect calorimetry. **f** Relative mRNA levels of the indicated hepatic proteins. **g** Levels of the indicated proteins in liver extracts from mice measured by IB with the LC3-II/I ratio and p62 band intensities shown below the blot (left), liver sections with LC3 detected by IHC (middle), and the number of LC3-II puncta/cell (right, *n* = 10 hepatocytes) (scale bar = 10 μm). Source data are provided as a Source Data file. **a**–**g** Values are presented as mean ± SD. Statistical significance was measured using the (**b**, **c**, **e** line graph, **f**, **g**) one-way or **d** two-way ANOVA with the Bonferroni post-test, and (**e**, bar graph) Student’s *t*-test. *P < 0.05, **P < 0.01, and NS statistically not significant.

FGF21 and its analogs have beneficial lowering lipid effects in obese animals and humans^26–28^. Knowing that JMJD3 promotes hepatic autophagy, including lipophagy (Fig. 2), and mitochondrial fatty acid oxidation^15^, and importantly, FGF21-induced autophagy is dependent on JMJD3 (Fig. 4), we next asked whether JMJD3 has a role in mediating the lipid-lowering effects of FGF21. Administration of FGF21 to high-fat diet (HFD) obese mice decreased body weight without significant changes in food intake and decreased the size of liver (Fig. 7a). Levels of hepatic lipids and TG (Fig. 7b) and long-chain acylcarnitine were decreased, and levels of serum ketone bodies (Fig. 7c) and glucose tolerance (Fig. 7d) were increased. O_2_ consumption and CO_2_ production (Fig. 7e, Supplementary Fig. 10) were increased, indicating increased energy expenditure. FGF21 treatment also increased autophagy gene expression and autophagy (Fig. 7f, g). Each of these FGF21-mediated effects was blunted in hepatic JMJD3-downregulated mice (Fig. 7a–g). These results demonstrate that JMJD3 is required for FGF21-induced autophagy and beneficial outcomes, particularly lowering lipids, in obese mice.

### Expression of KLB in fatty livers improves FGF21 signaling

Circulating FGF21 levels are highly elevated in non-alcoholic fatty liver disease (NAFLD) patients, as well as, in obese animals, suggestive of impaired FGF21 signaling^18,29,30^. Further, decreased autophagic flux and defective autophagy have been implicated in the development of NAFLD^5,6,31^. We, therefore, examined the effect of a HFD on FGF21 signaling and hepatic autophagy in mice.

**Fig. 8:**
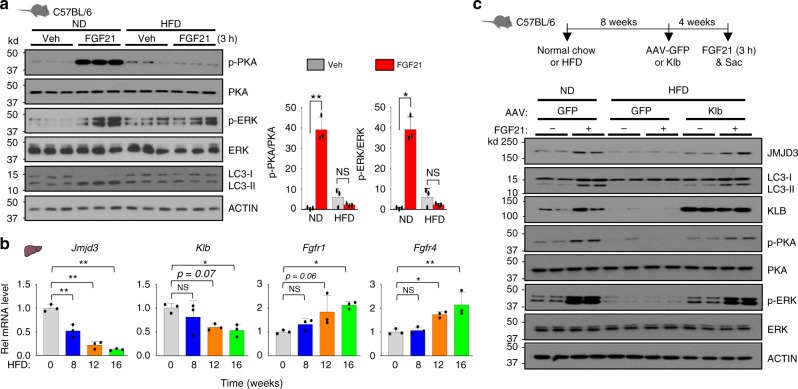
Expression of KLB in obese mice restores FGF21 signaling and autophagy. **a** Mice that had been fed a ND or HFD for 12 weeks were treated i.v. for 3 h with 0.1 mg/kg FGF21, and hepatic levels of the indicated proteins were detected by IB (left, *n* = 3 mice). The ratios of band intensities for the indicated proteins are shown (right)**. b** Mice were fed a HFD for the times indicated and mRNA levels of *Klb, JMJD3, Fgfr1, and Fgfr4* were measured by q-RTPCR (*n* = 3 mice). **c** Mice fed a HFD for 8 weeks were injected with AAV-TBG-KLB or AAV-TBG-GFP. Four weeks later, the mice were treated i.v. with vehicle or 0.1 mg/kg FGF21 for 3 h, and hepatic protein levels of the indicated proteins were detected by IB (n = 2 mice). Source data are provided as a Source Data file. **a**, **b** Values are presented as mean ± SD. Statistical significance was measured using the **a** two-way ANOVA with the Bonferroni or **b** one-way ANOVA with the Dunnett post-test *P < 0.05, **P < 0.01, and NS, statistically not significant.

Responsiveness to FGF21, as measured by increased p-PKA and p-ERK levels, was impaired, and FGF21-mediated induction of autophagy was absent in HFD obese mice compared to lean mice (Fig. 8a). Since β-Klotho (KLB) is the essential coreceptor for FGF21 action^18,19^, and its hepatic expression is downregulated in obesity^32^, we examined the effect of feeding a HFD for different lengths of time on expression of JMJD3, KLB, and FGFR. The mRNA levels of *JMJD3* and *KLB* were decreased, whereas those of *Fgfr1* and *Fgfr4* were increased, after feeding a HFD (Fig. 8b), suggesting that decreased expression of KLB may contribute to FGF21 resistance in obesity.

We, therefore, further tested whether restoring KLB levels in obese mice can rescue FGF21 signaling and subsequently, expression of JMJD3 and hepatic autophagy (Fig. 8c, top). Viral-mediated liver-specific expression of KLB in HFD obese mice resulted in increased p-ERK and p-PKA levels, increased JMJD3 protein levels, and increased ratios of LC3II/I proteins (Fig. 8c). These results suggest that restoring expression of KLB in obese mice improves FGF21 signaling and consequently, FGF21-induced autophagy.

### Expression of JMJD3 and autophagy genes is reduced in NAFLD

**Fig. 9:**
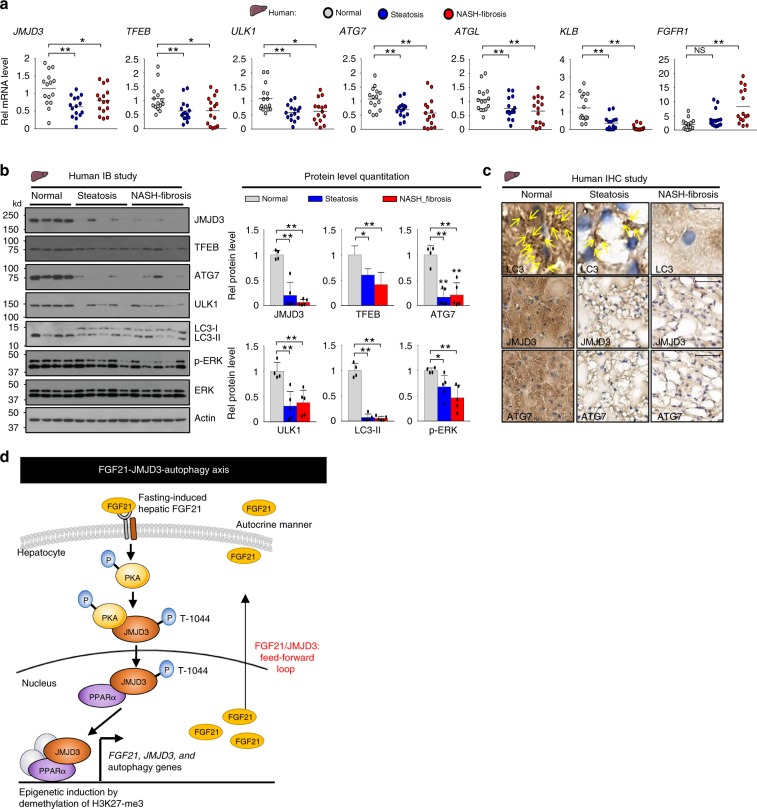
Expression of *JMJD3, TFEB*, and autophagy genes is reduced in NAFLD patients. Liver samples from normal, simple steatosis and NASH-fibrosis patients were analyzed. **a** The mRNA levels of the indicated genes measured by q-RTPCR (*n* = 15 individuals). **b** Levels of the indicated proteins in liver samples detected by IB (left, *n* = 4 normal, *n* = 5 patients, 3 to 4 pooled samples/lane) and quantified (right). **c** LC3, JMJD3, and ATG7 were detected in liver sections by IHC. LC3 puncta are indicated by yellow arrows (scale bar = 10 μm for LC3, 50 μm for JMJD3 and ATG7). Source data are provided as a Source Data file. **a**, **b** Values are presented as mean ± SD. Statistical significance was measured using the **a**, **b** one-way ANOVA with the Bonferroni post-test. *P < 0.05, **P < 0.01, and NS statistically not significant. **d** Model: in response to fasting, JMJD3 is phosphorylated at Thr-1044 by FGF21 signaling-activated PKA in mouse hepatocytes, which promotes its nuclear localization and interaction with the nuclear receptor PPARα to epigenetically induce transcription of genes involved in autophagy and lipid degradation, including *Atg7, Ulk1, Atgl, Tfeb, and Pgc-1α*, and as a positive feed-forward loop, *JMJD3 and FGF21* expression. Induced hepatic FGF21 sustains the activation of the FGF21-JMJD3-autophagy axis in an autocrine manner when fasting continues.

To assess potential human relevance of our findings, we examined the expression of JMJD3 and key autophagy genes in livers of human NAFLD patients. Autophagy has been reported to be defective in these patients^5,6,31^. Intriguingly, hepatic mRNA levels of *JMJD3, TFEB, ULK1, ATG7, and ATGL*, were all decreased in both simple steatosis and advanced NASH-fibrosis patients compared to normal subjects (Fig. 9a). The mRNA levels of *KLB* was also decreased in the patients (Fig. 9a) with decreased p-ERK levels, suggesting impaired FGF21 signaling^18,29,30^ (Fig. 9b). Protein levels of JMJD3, TFEB, ATG7, ULK1, LC3-II, and p-ERK, detected by IB of liver extracts (Fig. 9b) or IHC of liver sections (Fig. 9c), were decreased in the patients. While these results in humans are only correlative and do not necessarily link a defective FGF21-JMJD3 axis with defective autophagy in the patients, these findings are consistent with results in obese mice (Figs. 7 and 8), suggesting that the FGF21-JMJD3-autophagy axis is dysregulated in NAFLD.

## Discussion

The present study shows that histone demethylase JMJD3 is a key epigenetic activator of hepatic autophagy as part of a fasting-induced FGF21-JMJD3 signaling axis in mice. Numerous autophagy-network genes are induced by this axis under nutrient deprivation, including autophagy components, ATG7, LC3, and ULK1, autophagy gene activators, TFEB and Pgc-1α^7^, a lipase for autophagy-mediated lipid degradation, ATGL^17^, and expression of JMJD3 and FGF21, so that this axis likely has a major impact on autophagy. Since JMJD3 is conserved in many organisms^13,14^, including plants, yeast, C. elegans, mice, and humans, JMJD3-mediated epigenetic induction of autophagy might be a common adaptive mechanism for cellular survival and the maintenance of energy balance when nutrient deprivation persists.

Defective autophagy contributes to abnormal accumulation of hepatic TG and to fatty liver^5,6^ but the underlying mechanisms are not clearly understood. In this study, we identify a signaling mechanism by which JMJD3 promotes autophagy, including lipophagy, mediated by the fasting-induced hepatokine, FGF21. Impaired autophagy in FGF21-LKO mice strongly suggests that hepatic FGF21-induced physiologically during fasting can activate autophagy in an autocrine/paracrine manner. Remarkably, FGF21-mediated increases in autophagy and decreases in hepatic TG levels in mice were markedly attenuated by downregulation of hepatic JMJD3. Furthermore, JMJD3-mediated decreases in hepatic TG levels were largely abolished by liver-specific downregulation of *ATG7* in mice, suggesting the critical role of functional autophagy in regulation of lipid levels by JMJD3. Recently, JMJD3 was shown to promote browning of white adipose tissue upon cold exposure, thereby increasing energy expenditure^33^. It will be, thus, interesting to see whether JMJD3 is also activated in adipocytes by fasting-induced FGF21 signaling to regulate energy balance as is the case in the liver.

Nutrient deprivation in the liver promotes hydrolysis of TG from lipid droplets to supply free fatty acids for mitochondrial β-oxidation for energy production and substrates for ketone body formation. Recently, we have shown that JMJD3, together with the fasting-sensing factors, SIRT1 and PPARα, promotes fatty acid β-oxidation, and that liver-specific downregulation of JMJD3 led to fatty liver and glucose intolerance^15^. In the present study, we further show that JMJD3 promotes autophagy-mediated lipid degradation and that JMJD3 is important for FGF21-mediated beneficial effects on decreased liver TG levels and improved autophagy in obese mice. These findings, together, identify JMJD3 as a key epigenetic activator mediating hepatic fasting responses by inducing transcription of hepatic network genes involved in autophagy-mediated lipolysis and fatty acid β-oxidation to maintain energy balance.

Despite extensive studies on FGF21^18,19^, intracellular signaling mechanisms by which the FGF21 signal is transformed to epigenetic regulation of genes is poorly understood. The present study reveals that FGF21-activated PKA mediates the phosphorylation of JMJD3, which is important for its nuclear localization and activation of autophagy genes. In recent phosphoproteome studies in adipocytes, FGF21 treatment increased phosphorylation of numerous kinases in addition to ERK1/2, including PKA^34^, which supports our conclusion that PKA is a mediator of the FGF21-JMJD3-autophagy axis. Our studies in vivo, hepatocytes, and in vitro provide strong evidence that PKA-mediated phosphorylation of JMJD3 at Thr-1044 in response to FGF21 acts as a key epigenetic switch to activate JMJD3, which results in induction of autophagy genes by demethylation of histone H3K27-me3 (Model, Fig. 9d). The critical role of PKA in mediating the FGF21-JMJD3-autophagy axis was surprising since FGF21 signaling is usually through the ERK pathway^18,19^. Further, we observed that inhibition of ERK, like PKA, also blocked FGF21-mediated autophagy (Supplementary Fig. 11), but not phosphorylation of JMJD3 (Fig. 6i), suggesting that ERK either acts downstream of PKA or independently of PKA/JMJD3. Further studies will be required to fully understand the interaction between PKA and ERK in response to FGF21 signaling.

There is increasing evidence that the cellular response to nutrient deficiency involves intriguing feed-forward autoregulatory loops. Upon nutrient deprivation, TFEB, a key transcriptional activator of autophagy, increases expression of *Tfeb* itself and also of *Pgc-1α and Pparα*, transcriptional activators of *Tfeb*^7^. Recently, JMJD3, together with SIRT1 and PPAR*α*, was shown to form a positive autoregulatory loop upon fasting, and induces hepatic expression of their own genes, as well as, that of *Fgf21*^15^. In the present study, we further show that FGF21 activates JMJD3 via phosphorylation and in turn, this FGF21-activated JMJD3 induces expression of *Fgf21*. JMJD3 also epigenetically upregulates expression of its own gene and gene activators of autophagy, *Tfeb, Pgc-1α, Sirt1, and Pparα*. Thus, fasting triggers a positive feed-forward loop for autophagy induction wherein key components of the loop, including *Jmjd3, Fgf21, Tfeb, and Pparα*, positively regulate expression of each other to reinforce and amplify cellular responses to the fasting signal in the liver.

Circulating FGF21 levels are elevated in NAFLD patients^18,29,30^, suggestive of FGF21 resistance in obesity and defective autophagy has been implicated in the development and pathogenesis of NAFLD^5,6,31^. In the current study, we observed that hepatic expression of the FGF21 coreceptor^35^, KLB, is reduced in human NAFLD patients, as well as in obese mice, consistent with decreased FGF21 signaling. Remarkably, in vivo rescue of KLB expression in obese mice restored p-ERK and p-PKA levels, indicative of improved responsiveness to FGF21 signaling, and increased hepatic autophagy. We also observed that hepatic expression of autophagy components, ATG7, ULK1, and LC3-II, as well as autophagy gene activators, JMJD3 and TFEB, and p-ERK levels are decreased in NAFLD patients. Although correlative, these results suggest that a defective FGF21-JMJD3 axis may contribute to decreased autophagy flux in these patients, which has been reported previously^34^. Additional studies in humans will be required to determine whether there is a causal link between decreased FGF21 signaling and JMJD3 levels and decreased expression of autophagy genes and development of fatty liver in the patients.

Autophagy was shown to improve health and extend longevity^36,37^. Intriguingly, both JMJD3 and FGF21 extend life span in mice^14,38^, which potentially could be a result of their effects on promoting autophagy as shown in this study. Conversely, defective autophagy has been implicated in many diseases and aging^36,37^. Small molecule modulators targeting autophagy have been extensively studied as possible pharmacological agents for treatment of human diseases^39,40^. In this study, FGF21-induced phosphorylation of JMJD3 at Thr-1044 was important for induction of genes involved in hepatic autophagy and lipid degradation. Notably, such epigenetic regulators like JMJD3 often act in a gene-specific manner^15^ and posttranslational modifications of gene regulatory proteins may also modulate transcriptional outcomes in a gene-selective manner^16,41,42^. FGF21 signal-induced phosphorylation of JMJD3 at a single residue, Thr-1044, may, thus, provide a highly specific therapeutic option for treatment of NAFLD and other diseases associated with autophagy dysfunction.

## Methods

### Materials and reagents

Information on antibodies is provided in Supplemental Table 1. Rapamycin, bafilomycin A_1,_ WY14643, oleic acid, and inhibitors of ERK and PKA were purchased from Sigma Inc, ON-TARGET*plus* mouse siRNAs for PPARα (J-040740), CREB1 (J-040959), FOXO1 (J-041127), FGF21 (M-063178-01-0005), and JMJD3 (J-063799) from Dharmacon, Inc, adenoviruses for JMJD3 and shRNA for ATG7 and KLB AAV viruses from Vector Builder, and AAV-TBG-Cre from Vector Biolabs.

### Animal experiments

For liver-specific downregulation of JMJD3, 8-week-old male JMJD3-floxed mice^15^ were injected with AAV-TBG-Cre or -GFP (1.0 to 2.0 × 10^11^ active viral particles), and 12 weeks later the mice were fasted overnight or injected with FGF21 (0.1 mg/kg) 3 h before killing. For adenoviral expression of JMJD3, C57BL/6 mice were injected with Ad-GFP (control) or Ad-JMJD3 (0.5 to 1.0 × 10^9^ active viral particles) 4 weeks before killing. For hepatic expression of JMJD3 or shRNA for ATG7, C57BL/6 male mice were injected with Ad-JMJD3 or Ad-shATG7 for 4 weeks before killing. PPARα-KO (Jackson Lab) and FGF21-LKO mice^22^ were fasted for 24 h or fed with normal chow. For hepatic downregulation of JMJD3 in obese mice, male JMJD3-floxed mice fed a HFD (60% fat; Research Diets) for 4 weeks were injected with AAV-TBG-Cre or -GFP and administrated FGF21 (5 mg/kg, i.v.) once every 2 days for 4 weeks as reported^26^. For GTT, mice were fasted overnight and injected i.p. with 2 g/kg glucose, and blood glucose levels were measured using an Accu-Chek Aviva Glucometer (Roche), and liver TG levels were measured using a Kit (Abcam, ab65336). The metabolic rate was measured by indirect calorimetry using the Comprehensive Lab Animal Monitoring System (Columbus Instruments). Mice were housed individually and maintained at 23 °C with 12 h light/dark cycles. Food and water were available ad libitum and O_2_ consumption and CO_2_ production were measured. All animal use and viral protocols were approved by Institutional Animal Use and Care and Biosafety Committees at the University of Illinois at Urbana-Champaign (UIUC).

### Metabolomic analysis

For measuring acylcarnitine levels, liver samples (5 mg) were mixed with 500 μl of 90% of acetonitrile, sonicated for 10 s, and placed on ice for 20 min. After centrifugation at 14,000 rpm for 10 min, 100 μl of the supernatant was collected, and then, analyzed by using the Q-Exactive MS system (Thermo Fisher Scientific). Software Xcalibur 4.1.31.9 was used for data acquisition and analysis. The samples (15 μl) were injected into a Dionex Ultimate 3000 series HPLC system instrument in an isocratic flow (0.35 mL/min; 50% methanol in water with 0.1 % formic acid). Mass spectra were acquired under positive ESI (sheath gas flow rate, 49; aux gas flow rate: 12; sweep gas flow rate, 2; spray voltage, 3.5 kV; capillary temp, 259 °C; Aux gas heater temp, 419 °C) and the AGC target was 1E6 with a maximum injection time of 50 ms. The acylcarnitines were identified by the accurate mass. For measuring serum β-hydroxybutyrate level, 150 μl of acetonitrile was added to 50 μl of blood. Samples were vortexed, centrifuged at 14,000 rpm at 4 °C for 10 min, 100 μl of supernatant was transferred into a glass vial and derivatized by adding 50 μl of *N*-Trimethylsilyl-*N*-methyl trifluoroacetamide + 1% trimethylchlorosilane (Thermo Fischer Scientific) for 1 h at 50 °C, and then, injected into the GC-MS system consisting of Agilent 7890B gas chromatography and an Agilent 5977 A MSD. Separation performed on a ZB-5MS capillary column (Phenomenex). A constant flow rate of 2 ml/min for the helium carrier gas was maintained at 70 °C for 5 min, followed by increases of 5 °C/min to 120 °C, then 40 °C/min to 300 °C for 5 min. The mass spectrometer operated in positive electron impact mode at 69.9 eV ionization energy at m/z 33–600 scan range. Samples were analyzed in combined scan/SIM mode (m/z 233). β-hydroxybutyrate identification was performed using the mass spectra obtained from the authentic standards (Sigma-Aldrich) and additionally confirmed with NIST08 and W8N08 libraries (John Wiley & Sons, Inc.). A calibration curve was generated for the 10-0.15 mM concentration range. Quantitation was performed using Mass Hunter Quantitative Analysis B.08.00 (Agilent Inc.) software. LC-MS and GC-MS analyses were performed in the Metabolomics Laboratory of Roy J. Carver Biotechnology Center, University of Illinois at Urbana-Champaign.

### RNA-seq

JMJD3 was downregulated by infection of JMJD3-floxed mice with AAV-TBG-Cre, and controls were injected with AAV-GFP. The mRNAs from livers of fasted mice (*n* = 3/group) were prepared using the RNeasy mini prep kit (Qiagen). The cDNA libraries were sequenced using an Illumina HiSeq2000 (Illumina, San Diego, CA) to produce paired-end 100 bp reads. One library of reads per biological sample was examined for sequencing errors prior to mapping. Sequencing alignment was performed by STAR ver 2.5.0a. Gene ontology analysis was performed using the program DAVID.

### ChIP-seq

JMJD3-floxed mice were infected with AAV-TBG-Cre or AAV-GFP for 3 months and fasted for 16 h (*n* = 2/group). The precleared liver chromatin samples were immunoprecipitated with H3K27-me3 antibody, chromatin was eluted, and DNA was isolated. Samples containing 18 ng of DNA were used for genomic sequencing (Biotechnology Center, UIUC). Raw sequencing reads were processed using the AQUAS pipeline (https://github.com/kundajelab/chipseq_pipeline, git commit version id: 910ba91b9e9ba51f51497b39134c8e737a5184a5), which is based on the ENCODE (phase-3) histone ChIP-seq pipeline specifications. The project-specific parameters used were ‘-type histone -species mm10 –use_pooled_ctl’. Specifically, reads were mapped to the mouse reference genome (mm10) using bwa (version 0.7.13). Post-alignment filtering was done by Samtools (version 1.2) and Picard (version 1.126). Peak calling was done by MACS (version 2.1.0). The final overlapped peaks across replicates were determined as the H3K27-me3 enriched regions. Mouse gene annotation (refGene) for mm10 was downloaded from the UCSC Genome Browser. GO analysis was performed using DAVID (david.abcc.ncifcrf.gov).

### Mass spectrometry

Flag-mouse JMJD3 was expressed in PMH and 48 h later, the cells were treated with 5 µM MG132 for 4 h and then, treated with FGF21 (100 ng/ml) for 30 min. Flag-JMJD3 was purified by binding to M2 agarose, the beads were washed 10 times with IP buffer, and the bound proteins were analyzed by LC-MS/MS. Mass spec analyses were carried out using a Thermo LTQ Fusion Orbitrap connected to a Thermo Dionex 3000 nano RSLC. Chromatography was accomplished using a 15-cm Thermo Acclaim PepMap 100 C-18 column with mobile phase of 0.1% FA in water (A) and acetonitrile with 0.1% FA (B) at a flow rate of 300 nl/min and temperature at 40 °C. Peptides were eluted from 5% B to 60% B in 60 min. The mass spectrometer was operating in the data dependent mode, and precursor scans from 300 to 1500 m/z (120,000 resolution) were followed by collision (35% NCE, 1.6 m/z isolation window, 60 s exclusion window). Raw data were analyzed by in house Mascot Server (version 2.5.1) against the NCBIProt Mus musculus database 0829 (1 September 2019) containing 150,813 sequences. Search parameters were: peptide mass tolerance 10 ppm; fragment tolerance 0.6 Da; cleavage agent was trypsin; miscleaves 2,3,4; variable modifications were set for Acetyl (Protein-term), Oxidation (M), Phospho (ST), Phospho (Y); FDR 1%. Protein identification and PTM cutoff were set for *p* < 0.05. The *p* value for the identification and characterization of the phosphorylated JMJD3 peptide was calculated using Mascot^43^ (Matrix Science (London, UK), which uses probability based scoring.

### Transmission electron microscopy

Mouse liver samples were fixed in Karnovsky’s fixative and with 2% osmium tetroxide followed by the addition of 3% potassium ferricyanide for 30 min. After washing with water, samples were stained with uranyl acetate, dehydrated with ethanol, and the sample was embedded in epoxy using the Epon substitute Lx112. Ultrathin sections were stained with uranyl acetate and lead citrate, and imaged with a Hitachi H600 transmission electron microscopy (TEM).

### Immunofluorescence

Hepa1c1c7 cells were transfected with expression plasmids for GFP-LC3 and JMJD3 and 48 h later were incubated in complete media or HBSS for 2 h. Lipids were stained with BODIPY and counterstained with DAPI and were detected by confocal microscopy. For IHC, proteins in mouse liver were detected using an HRP/DAB kit (ab64261, Abcam). Nuclei were stained with hematoxylin, and samples were imaged with a Nanozoomer (Hamamatzu). Liver tissue was frozen in OCT compound, sectioned, and stained with H&E and Oil Red O.

### ChIP, re-ChIP, and CoIP

Liver tissue was minced, washed twice in PBS, and then incubated with 1% formaldehyde for 10 min at 37 °C. Glycine was added to 125 mM for 5 min at room temperature. Chromatin solutions in sonication buffer (50 mM Tris-HCl, pH 8.0, 2 mM EDTA, and 1% SDS) were sonicated four times with 10 s intervals using a QSonica XL-2000 instrument at power output setting 8. Then, chromatin sample was precleared and chromatin was immunoprecipitated using 1–2 µg of antibody or IgG. The immune complexes were collected by incubation with a Protein G Sepharose slurry for 1 h, washing with 0.1% SDS, 1% Triton X-100, 2 mM EDTA, 20 mM Tris-HCl, pH 8.0, three times containing successively 150 mM NaCl, 500 mM NaCl, or 0.25 M LiCl, and then eluted and incubated overnight at 65 °C to reverse the crosslinking. DNA was isolated for qPCR. For re-ChIP, chromatin samples were immunoprecipitated with the first antibody, the beads were washed, chromatin was eluted with 10 mM DTT, diluted 20X with 20 mM Tris-HCl, pH 8.0, 150 mM NaCl, 2 mM EDTA, 1% Triton X-100, and immunoprecipitated with the second antibody. Sequences of primers used for the qPCR are in Supplementary Table 2. For CoIP, cell extracts were prepared by brief sonication in CoIP buffer (50 mM Tris–HCl, pH 8.0, 150 mM NaCl, 2 mM EDTA, 0.5% NP‐40, 5% glycerol). The samples were incubated with 1-2 μg of antibodies for 3 h and 30 μl of a 25% protein G agarose slurry was added. One hour later, beads were washed with CoIP buffer three times and bound proteins were detected by IB.

### Cell culture

Primary mouse hepatocytes (PMH) were isolated by collagenase (0.8 mg/ml, Sigma, Inc) perfusion through the portal vein of mice anesthetized with isoflurane. Hepatocytes were filtered through a cell strainer (100 µm nylon, BD), washed with M199 medium, resuspended in M199 medium, centrifuged through 45% Percoll (Sigma, Inc.), and cultured in M199 medium containing 10% FBS. Hepa1c1c7 and HepG2 cells were cultured in DMEM containing 10% FBS.

### Luciferase reporter assays

DNA fragments near *Tfeb* and *Atg7* that contained PPARα peaks^9^ were amplified by PCR from mouse genomic DNA and cloned into the pGL3-basic vector (Promega). The DR1 motifs were mutated using site-directed mutagenesis (Agilent Tech). Hepa1c1c7 cells were transfected with indicated plasmids and luciferase activities were normalized to β-galactosidase activities.

### Nuclear localization studies

Nuclear and cytoplasmic fractions of the PMH cells were isolated using NE-PER Nuclear and Cytoplasmic Extraction Reagents (Thermo-Fisher Scientific Inc.) and the proteins were detected by IB. Cytoplasmic GAPDH and nuclear LAMIN-A were detected to assess the quality of the fractionation. For imaging studies, Hepa1c1c7 cells were fixed with 4% paraformaldehyde, permeabilized with PBS containing 3% BSA, 0.1% Triton X-100, incubated with M2 antibody for 2 h, washed and incubated with Alexa Fluor 488-conjugated donkey anti-mouse IgG for 1 h. Nuclei were stained with Hoechst 33,258 and imaged by confocal microscopy (Zeiss, LSM700).

### In vitro kinase assay

Flag-JMJD3 proteins expressed in HepG2 cells were isolated by M2-agarose, the beads were washed with the lysis buffer and with kinase buffer (50 mM Tris-HCl, pH 7.5, 10 mM MgCl2, 1 mM DTT). Flag-proteins bound to M2-agarose were incubated with 20 μM ATP and 20 ng of PKA (Biorbyt) or ERK1/2 (MyBiosource) in kinase buffer at 30 °C for 30 min, and JMJD3 phosphorylation at Thr was detected by IB.

### GST pull down assay

Bacterially expressed and affinity purified GST-JMJD3 proteins^15^ were incubated with Pka or ERK1/2, and bound proteins were detected by IB.

### Quantification of mRNA

RNA was isolated from liver and quantified by q-RTPCR, normalized to 36b4 mRNA. Primer sequences are in Supplementary Table 2.

### NAFLD patient study

Liver specimens from 15 unidentifiable normal individuals or steatosis or severe NASH-fibrosis patients were obtained from the Liver Tissue Procurement and Distribution System that operates under a contract from the National Institutes of Health. Because the specimens or data were not collected specifically for this study and no one on our study team has access to the subject identifiers linked to the specimens or data, this study is not considered human subjects research and ethical approval was not required (See §46.104 in Part 46—Protection Of Human Subjects in the Electronic Code of Federal Regulations at the following link: https://www.ecfr.gov/cgi-bin/retrieveECFR?gp= &SID=83cd09e1c0f5c6937cd9d7513160fc3f&pitd=20180719&n=pt45.1.46&r=PART&ty=HTML#se45.1.46_1104). Protein levels were detected by IB and IHC and mRNA levels were quantified by q-RTPCR.

### Statistical analyses

GraphPad Prism 6 (GraphPad software version 6.01) was used for data analysis. Statistical significance was determined by the Mann–Whitney test or one- or two-way ANOVA with the Bonferroni post-test for single or multiple comparisons as appropriate. Whenever relevant, the assumptions of normality were verified using the Shapiro–Wilk test, Kolmogorov-Smirnov test and the D’agostino-Pearson omnibus test. *P*-values < 0.05 were considered as statistically significant.

### Reporting summary

Further information on research design is available in the Nature Research Reporting Summary linked to this article.

## Supplementary information

Supplementary Information

Reporting Summary

## Data Availability

The data that support the findings of this study are available from the corresponding author upon reasonable request. The RNA-seq and ChIP-seq data are deposited in the GEO database with the Accession Numbers GSE137555 [https://www.ncbi.nlm.nih.gov/geo/query/acc.cgi?acc=GSE137555] and GSE138157 [https://www.ncbi.nlm.nih.gov/geo/query/acc.cgi?acc=GSE138157], respectively. The mass spectrometry proteomics data have been deposited to the ProteomeXchange Consortium via the PRIDE partner repository (http://www.ebi.ac.uk/pride) with the dataset identifier PXD015672. The source data underlying Figs. 1a, e–g, 2a–e, g, 3a–h, 4a–f, 5b–g, 6b, d–I, 7a–h, 8a–c, and 9a, b and Supplementary Figs. 1a–c, 3a–f, 5, 6a–c, 7a, c–f, 8b–g, 9b, c, 10, and 11 are provided as a Source Data file.

## Source Data

Source Data
