# Supplementary material for: Fasting-induced FGF21 signaling activates hepatic autophagy and lipid degradation via JMJD3 histone demethylase
Source: Nat Commun. 2020 Feb 10;11:807. doi: 10.1038/s41467-020-14384-z (PMC7010817; doi:10.1038/s41467-020-14384-z)
Supplement: Supplementary file 1 — Supplementary Information [file 41467_2020_14384_MOESM1_ESM.pdf]

Supplementary Information

Fasting-induced FGF21 signaling activates hepatic autophagy and lipid degradation via JMJD3 histone demethylase

Byun et al.

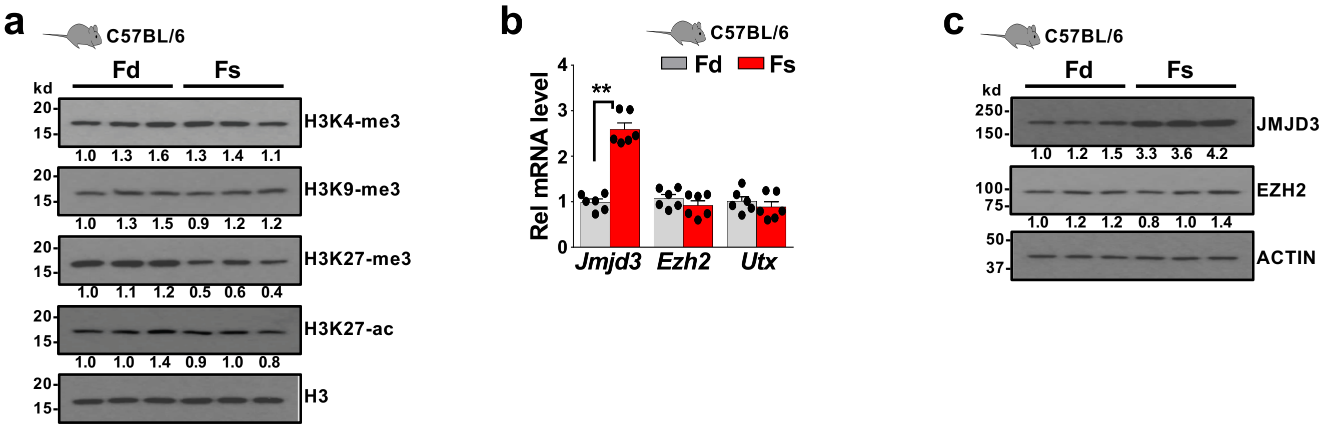

Supplementary Figure 1. Effects of fasting or feeding on levels of selected histone marks and H3K27-me3 modifying proteins in mouse liver. Male C57BL/6 male mice were fasted (Fs) for 16 h or refed (Fd) for 16 h after fasting. **(a)** Levels of the indicated modified histones in liver extracts determined by IB. **(b, c)** The mRNA and protein levels of the indicated histone modifiers determined by q-RTPCR (n= 6) and IB (n=3), respectively. **(a,c)** Band intensities relative those in the first lanes are shown below the blots. **(b)** Values are presented as mean ± SD. Statistical significance was measured using the Mann-Whitney test. **P<0.01.

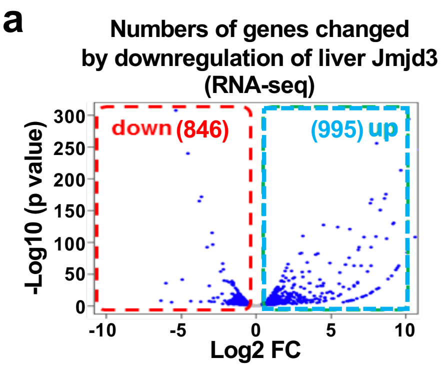

b

G/O analysis of up-regulated genes (↓ Liver Jmjd3) : 995 genes

GO ID	Gene Ontology Term	p-Value
GO:0007155	cell adhesion	1.43E-09
GO:0015629	actin cytoskeleton	1.34E-08
GO:0035556	intracellular signal transduction	1.53E-06
GO:0030154	cell differentiation	3.32E-06
GO:0001525	angiogenesis	8.60E-06
GO:0007275	multicellular organism development	8.89E-06
GO:0008285	negative regulation of cell proliferation	5.09E-04
GO:0001558	regulation of cell growth	9.77E-04
GO:0045721	negative regulation of gluconeogenesis	3.60E-03
GO:0016477	cell migration	5.16E-03

G/O analysis of down-regulated genes (↓ Liver Jmjd3) : 846 genes

GO ID	Gene Ontology Term	p-Value
GO:0016020	membrane	4.11E-13
GO:0006955	immune response	1.68E-11
GO:0031410	cytoplasmic vesicle	1.29E-05
GO:0005764	lysosome	2.25E-04
GO:0001561	fatty acid oxidation	4.46E-04
GO:0006914	autophagy	5.24E-04
GO:0006629	lipid metabolic process	6.81E-04
GO:0060612	adipose tissue development	1.24E-03
GO:0007623	circadian rhythm	5.75E-03

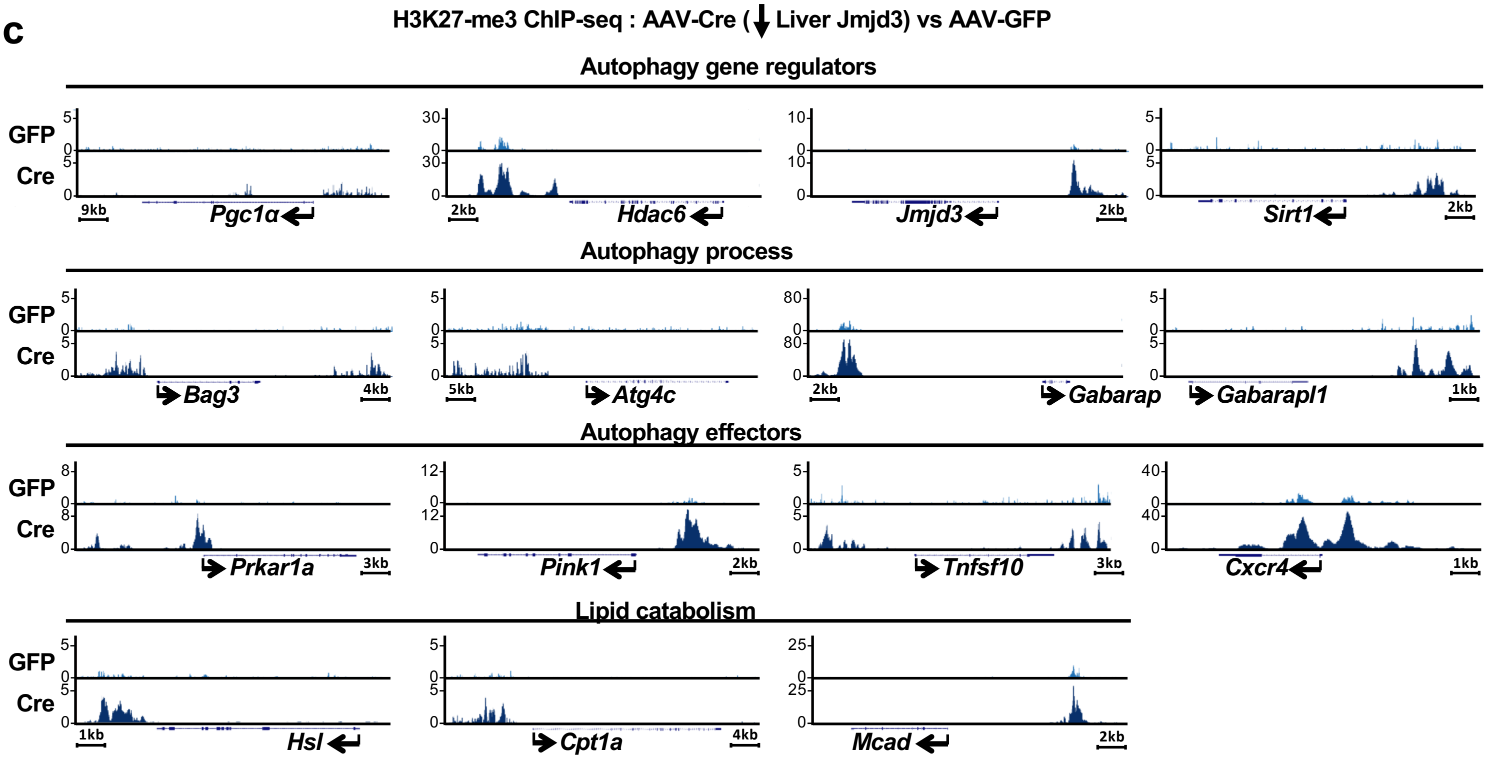

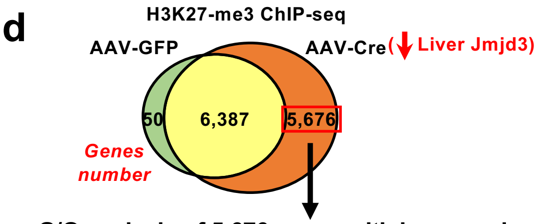

G/O analysis of 5,676 genes with increased H3K27-me3 (↓ Liver Jmjd3)

GO ID	Gene Ontology Term	p-Value
GO:0006955	immune response	4.62E-11
GO:0016298	lipase activity	3.26E-08
GO:0006629	lipid metabolic process	1.02E-07
GO:0006914	autophagy	1.18E-06
GO:0031410	cytoplasmic vesicle	1.90E-06
GO:0048583	regulation of response to stimulus	8.20E-06
GO:0022900	electron transport chain	8.82E-05
GO:0005746	mitochondrial respiratory chain	2.94E-04
GO:0005764	lysosome	6.96E-04
GO:0010506	regulation of autophagy	8.67E-04

Supplementary Figure 2. Effects of liver-specific downregulation of Jmjd3 on global gene expression and H3K27-me3 levels. Jmjd3 floxed mice were infected with hepatocyte-targeting AAV-TBG-Cre or control AAV-GFP for 12 weeks and then fasted for 16 h. RNA-seq and H3K27-me3 ChIP-seq analyses of liver were done in parallel. **(a)** RNA-seq: Volcano plots with the number of hepatic genes up- or down-regulated by 1.5-fold or more and **(b)** G/O analyses of hepatic genes up- (left) or down-regulated (right) in livers of Jmjd3-depleted mice compared to control mice. **(c)** Normalized H3K27-me3 peaks at selected genes involved in autophagic pathways as displayed by the UCSC genome browser. **(d)** Venn diagrams (top) showing the number of hepatic genes marked by H3K27-me3 peaks within ± 15 kb of the transcriptional start site in livers of control mice and liver–specific Jmjd3-downregulated mice. G/O analysis (bottom) of 5,676 genes with increased H3K27-me3 levels specifically in Jmjd3-downregulated mice.

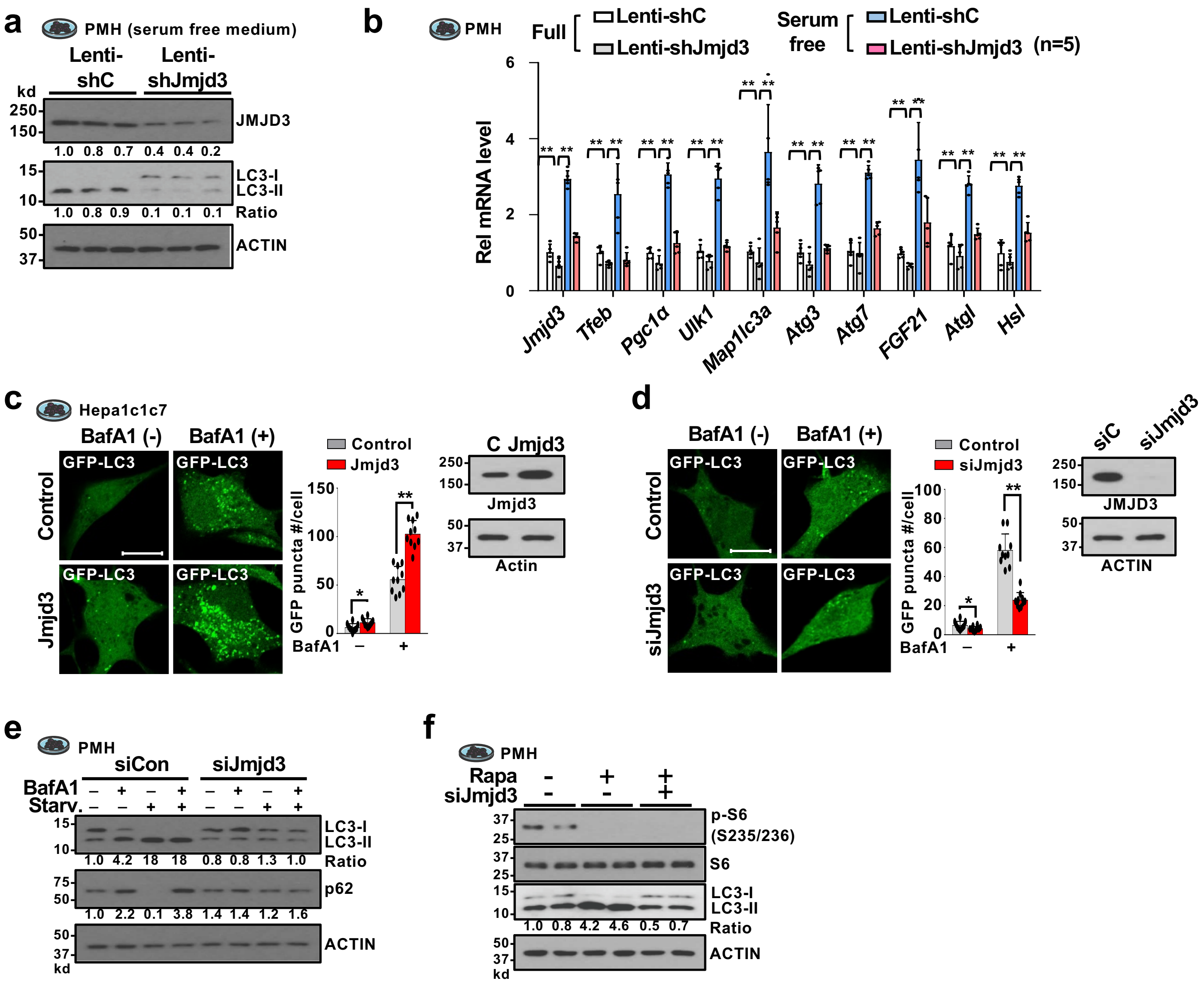

Supplementary Figure 3. Effects of bafilomycin A1 and rapamycin on the Jmjd3-induced autophagy. (a,b) PMH were infected with control lentivirus (shC) or lentivirus expressing Jmjd3 shRNA (shJmjd3) for 72 h in M199 full medium or serum-free medium. **(a)** Levels of JMJD3 and LC3 proteins in liver were determined by IB. Relative band intensities for JMJD3 and the LC3-II/I ratios are shown below the blots. **(b)** The mRNA levels of indicated genes were measured by q-RTPCR (n= 5). **(c,d)** Hepa1c1c7 cells were transiently transfected with expression vectors for **(c)** GFP-LC3 and control or Jmjd3 or **(d)** control RNA or Jmjd3 siRNA**.** The cells were cultured in serum free medium for 12 h, and treated with vehicle or 200 nM bafilomycin A1 (BafA1) for 2 h. Representative confocal images of cells (left) and the average number of LC3-II puncta/cell (n=10) (middle) and levels of JMJD3 and ACTIN (right) determined by IB are shown (scale bar=5 μm). **(e)** PMH were transfected with control siRNA (siCon) or Jmjd3 siRNA and treated with vehicle or 200 nM BafA1 for 2 h in complete M199 medium or HBSS starvation media (Starv.). The indicated proteins were detected by IB. The ratios of the LC3-II/I band intensities and the band intensities of p62 relative to those in the first lane are shown below the blots. **(f)** PMH were transfected with control or Jmjd3 siRNA, and 72 h later, cells were cultured in serum free M199 medium for 12 h and treated with 0.25 mg/ml of rapamycin (Rapa) for 4 h. The indicated proteins were detected by IB. Duplicates are shown. The ratios of the LC3-II/I band intensities are shown below the blots. **(b-d)** Values are presented as mean ± SD. Statistical significance was measured using the **(b-d)** two-way ANOVA with the Bonferroni post-test. *P<0.05, **P<0.01.

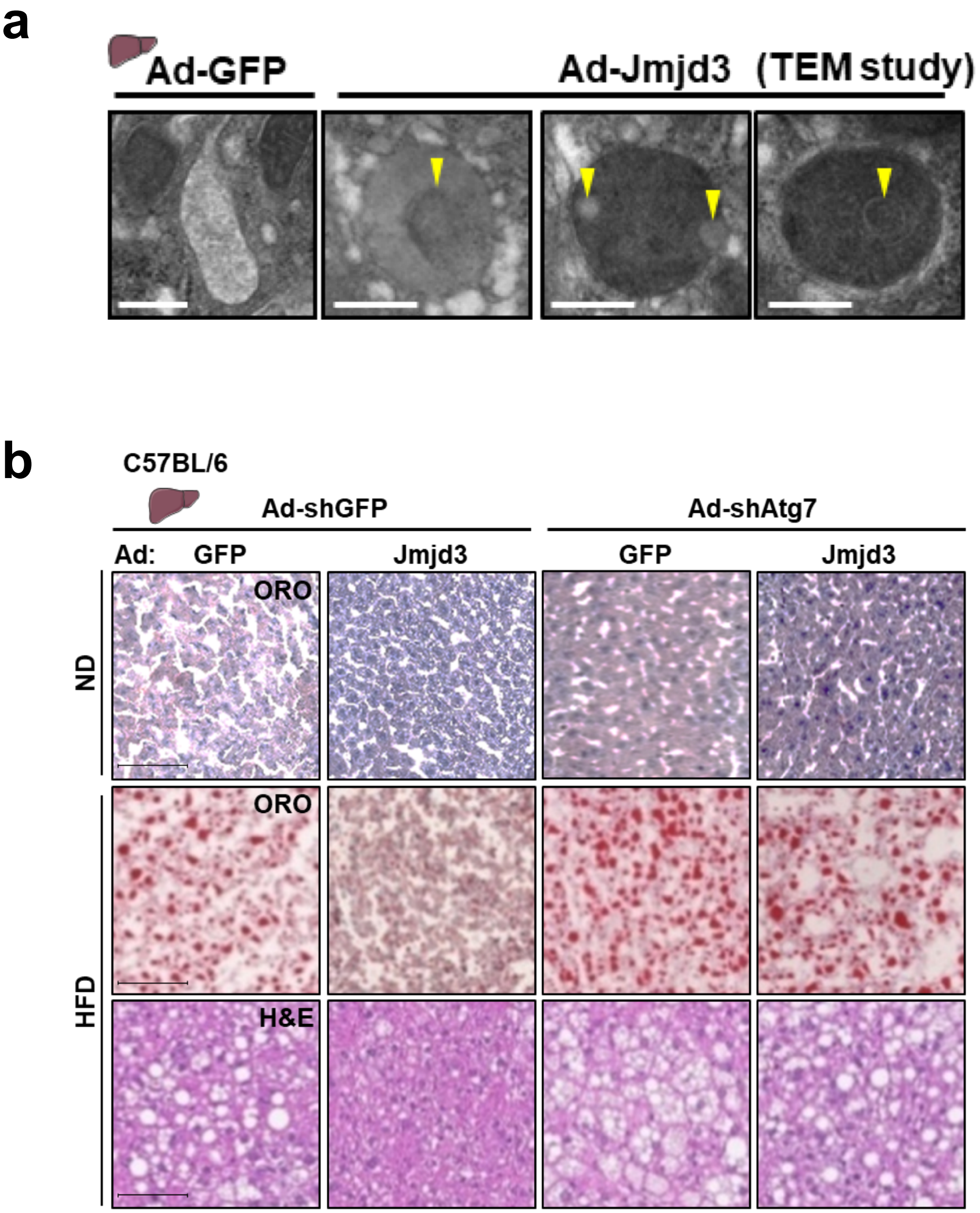

Supplementary Figure 4. (a) GFP (control) or Jmjd3 were adenovirally expressed for 4 weeks in C57BL/6 mice and mice were fasted for 8 h and then, liver sections were imaged by transmission electron microscopy Autophagy vesicles inside lipid droplets are indicated by the yellow arrowheads (scale bar=0.5 μm). **(b)** An enlarged version of the image in Fig. 2f showing Oil Red O (ORO) and H&E staining of liver sections (scale bar=50 μm).

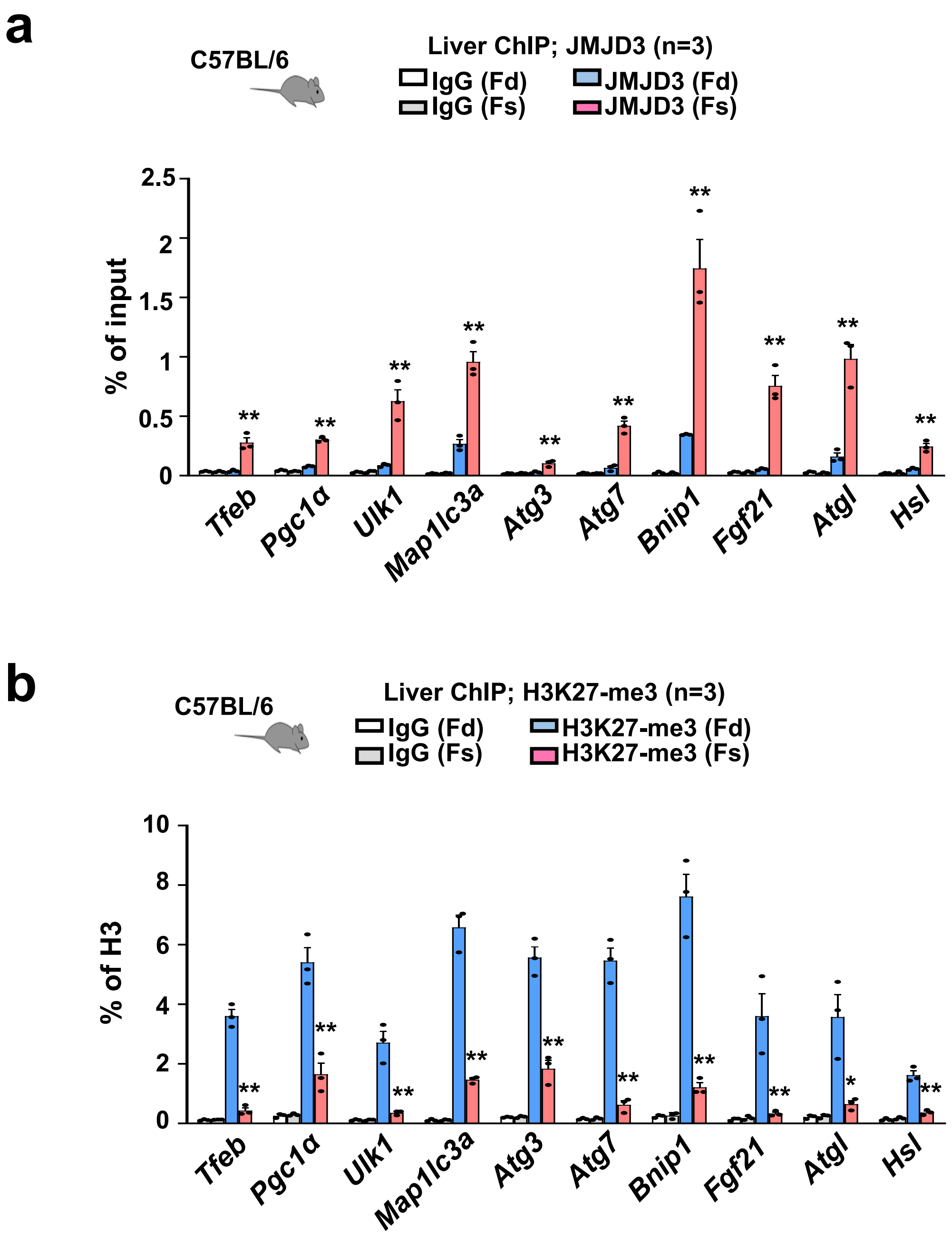

Supplementary Figure 5. Effects of fasting and feeding on occupancy of JMJD3 and histone H3K27-me3 levels at autophagy-promoting genes. ChIP: Mice were fasted overnight and then, refed for 6 h and liver ChIP assays were done to examine occupancy of JMJD3 proteins (a) and H3K27-me3 levels (b) at potential JMJD3 target genes involved in autophagy (n=3 mice). All values are presented as mean ± SD. Statistical significance was measured using the two-way ANOVA with the Bonferroni post-test. *P<0.05 and **P<0.01.

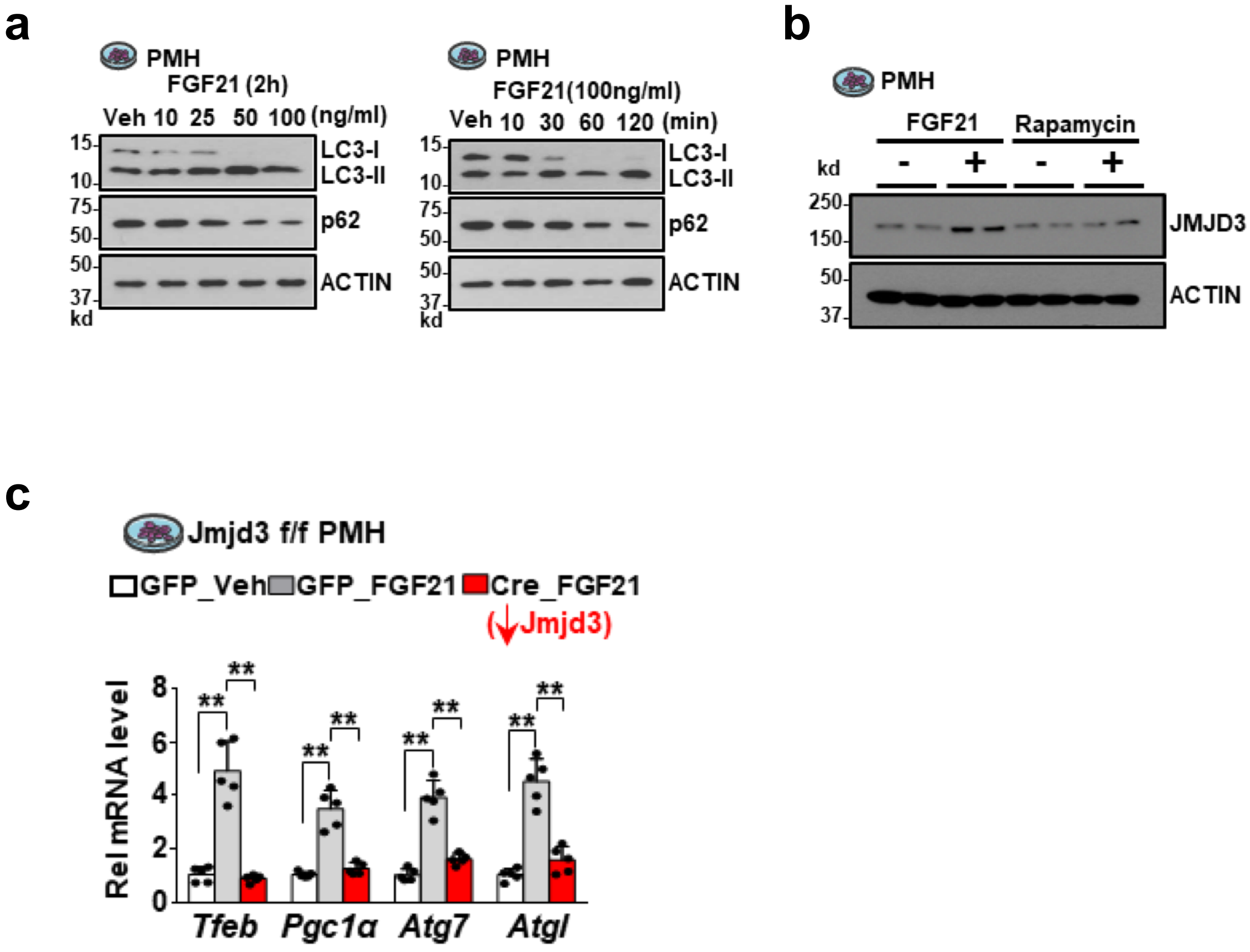

Supplementary Figure 6. FGF21-induced autophagy is likely mTOR-independent. **(a)** PMH were treated with vehicle or the indicated concentration of FGF21 for 2 h (left) or for different times as indicated with 100 ng/ml of FGF21 (right). The indicated proteins were detected by IB. **(b)** PMH were treated with 100 ng/ml of FGF21 or 0.25 mg/ml of rapamycin (Rapa) for 4 h. JMJD3 was detected by IB. Duplicates are shown. **(c) FGF21-mediated induction of autophagy genes is blunted after Jmjd3 downregulation in PMH.** Primary hepatocytes from Jmjd3 floxed mice were infected with AAV-TBG-GFP or AAV-TBG-Cre for 72 h, and treated with vehicle or FGF21 (100 ng/ml) for 12 h. The mRNA levels of hepatic autophagy-related genes measured by q-RTPCR (n=5). **(c)** All values are presented as mean ± SD. Statistical significance was measured using the one-way ANOVA with the Bonferroni post-test. $^{**}P<0.01$.

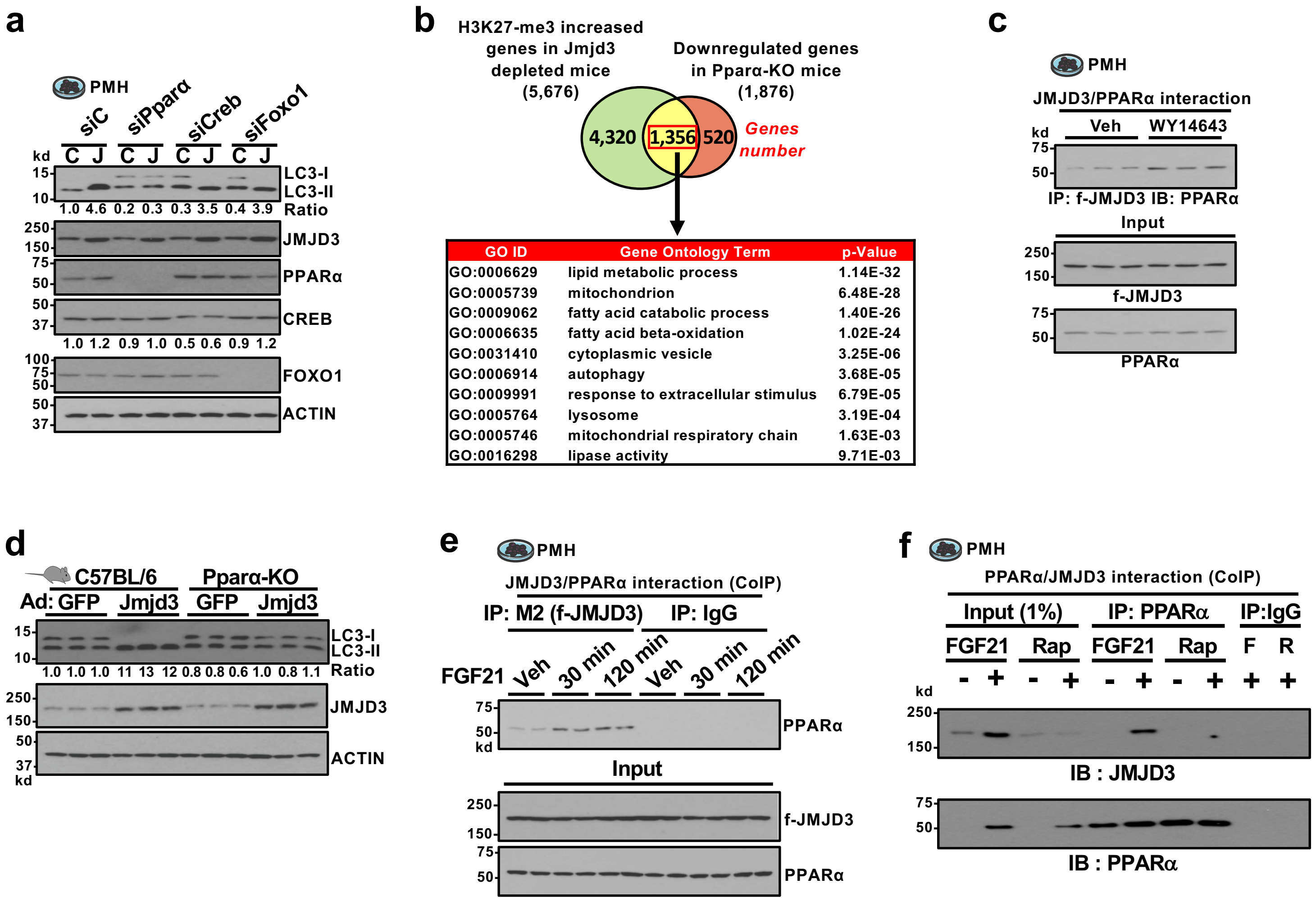

Supplementary Figure 7. PPARα is a key component of the FGF21-JMJD3-autophagy axis. (a-c) Genomic analyses reveal a potential role of PPARα in JMJD3-mediated hepatic autophagy. (a) PMH were cotransfected with an expression plasmid for Jmjd3 (J) or control plasmid (C) and with siRNAs and incubated in serum-free M199 medium overnight. Levels of the indicated proteins were determined by IB. The ratios of the LC3-II/LC3-I and band intensities for CREB1 are shown below the blots. **(b)** Venn diagram (top) for genes with increased H3K27-me3 levels in Jmjd3-downregulated mice (as shown in Supplementary Fig. 2d) and genes downregulated in Pparα-KO mice. G/O analysis (bottom) of the overlapping genes. **(c)** Jmjd3 was expressed in PMH from C57BL/6 mice for 48 h and the cells were treated with WY14643 (50 mM) for 3 h. PPARα in anti-flag immunoprecipitates and input proteins were detected by IB (n=3).

(d) JMJD3-mediated hepatic autophagy is blunted in Pparα-KO mice. C57BL/6 or Pparα-KO mice were injected with Ad-GFP or Ad-Jmjd3. After 4 weeks, the mice were fasted for 4 h before sacrifice. Hepatic LC3 levels were measured by IB. The ratios of the LC3-II/LC3-I band intensities relative to that in the first lane are indicated below the blot (n=3).

(e, f) FGF21 signaling induces the interaction of JMJD3 with PPARα. (e) CoIP analysis of the interaction of JMJD3 and PPARα. PMH were transfected with an expression vector for flag-Jmjd3 and 48 h later, cells were treated with 100 ng/ml FGF21 for the times indicated. PPARα in flag-JMJD3 (M2) or control IgG immunoprecipitates and input proteins were detected by IB (n=2). **(f)** CoIP analysis of the interaction of JMJD3 and PPARα. PMH were treated with FGF21 or rapamycin (0.25 mg/ml) for 4 h. JMJD3 levels in PPARα or control IgG immunoprecipitates and input proteins were detected by IB.

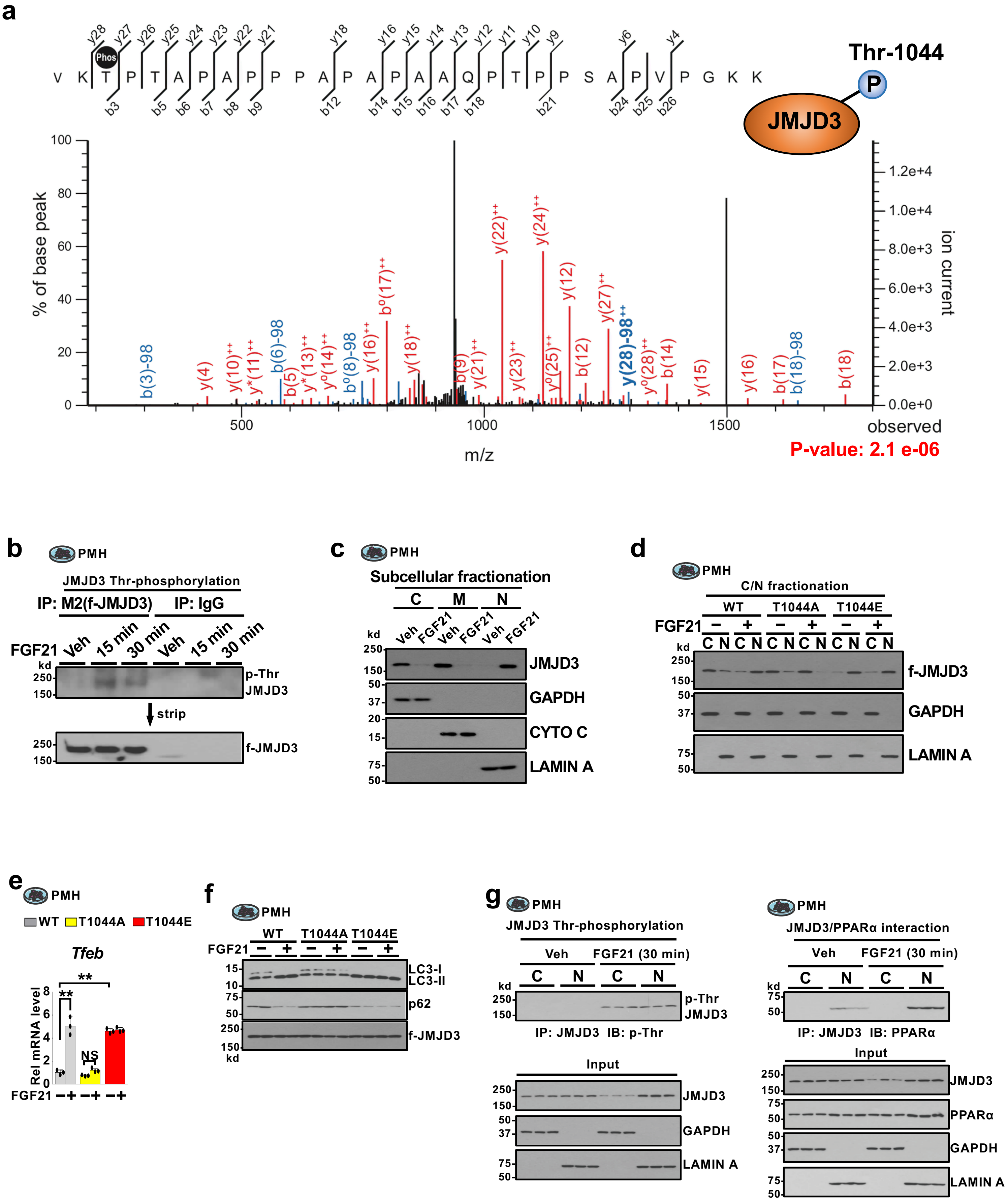
a
V K T P T A P A P P P A P A P A A Q P T P P S A P V P G K K
Phos
y28 y27 y26 y25 y24 y23 y22 y21 y18 y16 y15 y14 y13 y12 y11 y10 y9 y6 y4
b3 b5 b6 b7 b8 b9 b12 b14 b15 b16 b17 b18 b21 b24 b25 b26
Thr-1044
JMJD3
P
% of base peak
ion current
m/z
observed
P-value: 2.1 e-06
b(3)-98
y(4)
y(10)++
y*(11)++
b(6)-98
b(5)
y*(13)++
y°(14)++
b°(8)-98
y(16)++
b°(17)++
y(18)++
b(9)
y(21)++
y(22)++
y(23)++
y(24)++
y°(25)++
y(12)
b(12)
y(27)++
y(28)-98++
y°(28)++
b(14)
y(15)
y(16)
b(17)
b(18)-98
b(18)
b
PMH
JMJD3 Thr-phosphorylation
IP: M2(f-JMJD3)
IP: IgG
FGF21
Veh
15 min
30 min
kd
p-Thr JMJD3
strip
f-JMJD3
c
PMH
Subcellular fractionation
C M N
Veh FGF21
JMJD3
GAPDH
CYTO C
LAMIN A
d
PMH
C/N fractionation
WT T1044A T1044E
FGF21 – +
C N
f-JMJD3
GAPDH
LAMIN A
e
PMH
WT T1044A T1044E
Tfeb
Rel mRNA level
**
NS
FGF21 –+ –+ –+
f
PMH
WT T1044A T1044E
FGF21 – +
LC3-I
LC3-II
p62
f-JMJD3
g
PMH
JMJD3 Thr-phosphorylation
Veh
FGF21 (30 min)
C N
p-Thr JMJD3
IP: JMJD3 IB: p-Thr
Input
JMJD3
GAPDH
LAMIN A
JMJD3/PPARα interaction
IP: JMJD3 IB: PPARα
PPARα

Supplementary Figure 8. Phosphorylation of JMJD3 at T1044 is critical for its nuclear localization and induction of autophagy.

(a) An enlarged image of Fig. 6a. **(b)** Mouse flag-Jmjd3 was expressed in PMH and 48 h later, cells were treated with vehicle for 30 min or with 100 ng/ml FGF21 for the indicated times. Levels of Thr-phosphorylated JMJD3 (top) were detected by IP/IB, and flag-JMJD3 levels after stripping the membrane (bottom) were detected by IB. **(c)** PMH were treated with 0.1 mg/kg FGF21 for 3 h. Nuclear (N) fractions were isolated by centrifugation from PMH extracts, mitochondrial (M) fractions were isolated by further centrifugation, and then, the supernatant was cytoplasmic (C) fractions. JMJD3 and the cytoplasmic marker, GAPDH, mitochondrial marker, cytochrome C (CYTO C), and nuclear marker, LAMIN A, were detected by IB. **(d)** PMH were transfected with expression plasmids for flag-Jmjd3-WT or flag-Jmjd3-T1044A or -T1044E, as indicated, and after 48 h, cells were treated with vehicle or FGF21 (100 ng/ml) for 30 min. Nuclear (N) and cytoplasmic (C) fractions were isolated from cell extracts. Input JMJD3 and the cytoplasmic marker, GAPDH, and nuclear marker, LAMIN A, were detected by IB. **(e, f)** PMH were transfected with expression plasmids for WT-Jmjd3 and mutants as indicated, and after 48 h, cells were treated with vehicle or 100 ng/ml FGF21 for 2 h. **(e)** mRNA levels of indicated genes were measured by RT-qPCR (n=3) and **(f)** levels of LC3 and p62 were measured by IB. **(g)** PMH from C57BL/6 mice were treated with vehicle or FGF21 for 30 min and nuclear (N) and cytoplasmic (C) fractions were isolated. **(left)** p-Thr in anti-JMJD3 immunoprecipitates (top) and input protein levels were detected by IB (n=3). **(right)** CoIP: PPARα in anti-JMJD3 immunoprecipitates (top) and input protein levels as indicated were detected by IB (n=3). **(e)** Values are presented as mean ± SD. Statistical significance was measured using the two-way ANOVA with the Bonferroni post-test. **P<0.01, and NS, statistically not significant.

a

Position in query protein	Sequence in query protein	Corresponding motif described in the literature (phosphorylated residues in red)	Features of motif described in the literature
1039-1044	VSKVKT	[M/I/L/V]X[R/K]XX[pS/pT]	Chk kinase substrate motif
1039-1046	VSKVKTPT	[M/V/L/I/F]X[R/K]XX[pS/pT]XX	Calmodulin-dependent protein kinase II substrate motif
1040-1044	SKVKT	pSXXX[pS/pT]	MAPKAPK2 kinase substrate motif
1041-1044	KVKT	KXX[pS/pT]	PKA kinase substrate motif
1041-1044	KVKT	[R/K]XX[pS/pT]	PKC kinase substrate motif
1043-1045	KTP	X[pS/pT]P	ERK1, ERK2 substrate motif

b

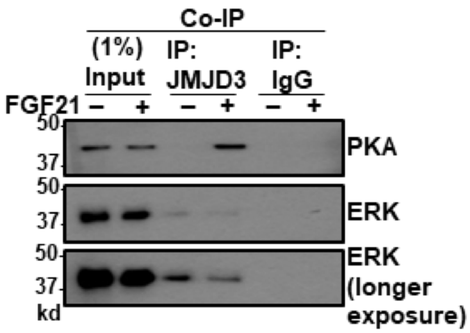

c

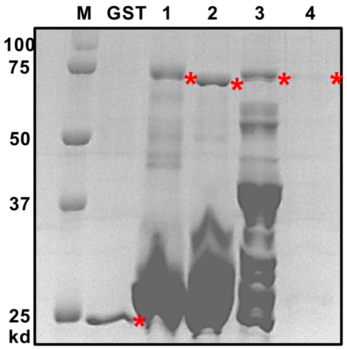

(Coomassie blue Staining)

Supplementary Figure 9. FGF21-activated PKA mediates the JMJD3 phosphorylation. (a) Prediction of possible phosphorylation sites and kinase motifs using the PhosphoMotif Finder program of the Human Protein Reference Database program (http://www.hprd.org). **(b)** CoIP: C57BL/6 mice were treated with 0.1 mg/kg FGF21 for 3 h before sacrifice. Either PKA or ERK in anti-JMJD3 immunoprecipitates (top) from liver extracts and input protein levels, as indicated, were detected by IB. **(c)** GST-JMJD3 fusion proteins used in GST pull down studies were visualized by Coomassie blue staining.

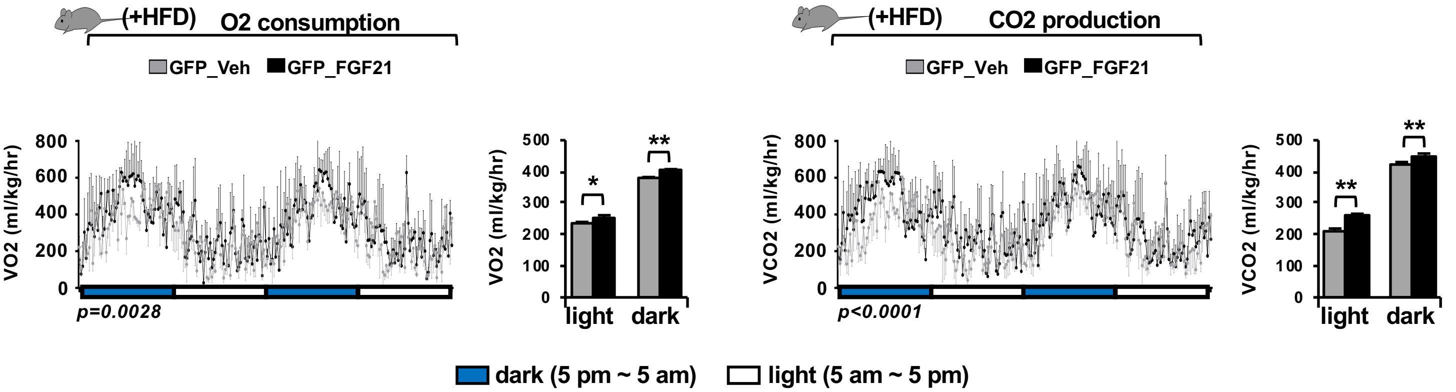

Supplementary Figure 10. Administration of FGF21 increases O_2 consumption and CO_2 production in obese mice. Jmjd3 floxed mice were fed a HFD for 4 weeks, injected with AAV-TBG-GFP control viruses, and then treated with vehicle or FGF21 once every 2 days for 4 weeks with continued feeding of a HFD (n=5 mice/group). Then, O_2 consumption and CO_2 production rates measured by indirect calorimetry. Values are presented as mean ± SD. Statistical significance was measured using the one-way ANOVA with the Bonferroni post-test **(line graph)** and Student's t-test **(bar graph)**. *P<0.05, **P<0.01.

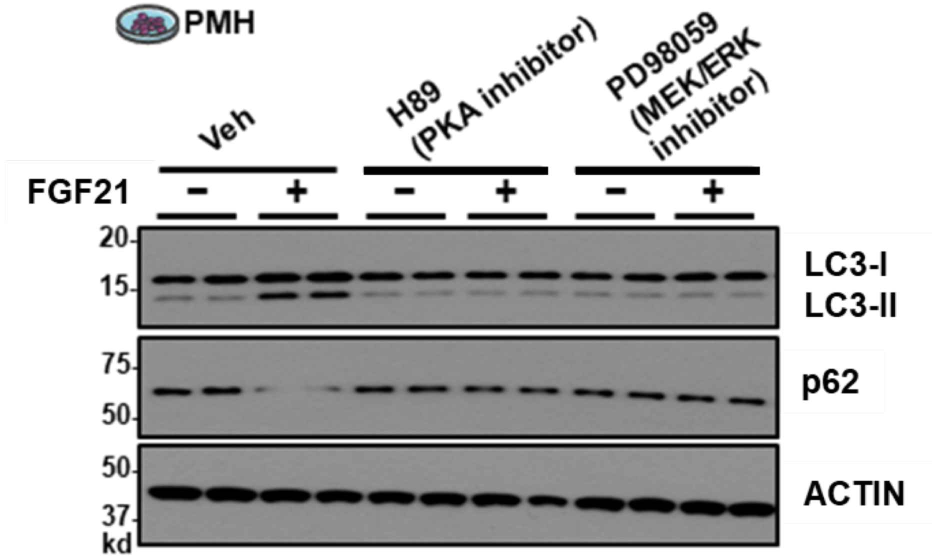

Supplementary Figure 11. Treatment of an inhibitor of either PKA or MEK/ERK resulted in inhibition of autophagy in hepatocytes. PMH were treated with FGF21 and with vehicle or PKA (H89, 10 μM) or MEK/ERK (PD98059, 40 μM) inhibitor for 30 min. Protein levels of ratios of LC3 II/I and p62 were detected by IB.

Supplementary Table1. Information on antibodies

Antibody	Company	# of catalogue	dilutions
JMJD3	Santa Cruz Biotechnology	sc-130159	1: 5000
KLB	Santa Cruz Biotechnology	sc-74343	1: 5000
GAPDH	Santa Cruz Biotechnology	sc-166574	1: 5000
LAMIN A	Santa Cruz Biotechnology	sc-20680	1: 5000
PPARα	Santa Cruz Biotechnology	sc-9000	1: 5000
LAMP1	Abcam	ab24170	1: 5000
H3K27-me3	Abcam	ab6002	1: 5000
H3K9-me3	Abcam	ab8898	1: 5000
H3K27-ac	Abcam	ab4729	1: 5000
histone H3	Abcam	ab1791	1: 10000
H3K4-me3	Abcam	ab8580	1: 5000
ACTIN	Cell Signaling	#4970	1: 10000
LC3-I/II	Cell Signaling	#4108	1: 3000
p62	Cell Signaling	#5114	1: 2000
TFEB	Cell Signaling	#37785	1: 3000
ULK1	Cell Signaling	#8054	1: 5000
p-Thr	Cell Signaling	#9386	1: 5000
EZH2	Cell Signaling	#5246	1: 3000
p-S6	Cell Signaling	#4858	1: 3000
S6	Cell Signaling	#2217	1: 5000
p-ERK	Cell Signaling	#9101	1: 3000
ERK	Cell Signaling	#4695	1: 5000
CREB	Cell Signaling	#9104	1: 3000
FOXO1	Cell Signaling	#2880	1: 5000
p-PKA	Cell Signaling	#4781	1: 5000
PKA	Cell Signaling	#4782	1: 5000
ATG7	Cell Signaling	#2631	1: 3000
KLB	R&D Systems	AF2619	1: 3000
FGF21	R&D Systems	AF3057	1: 3000
LAMP2	Sigma Inc	L0668	1: 5000
FLAG	Sigma Inc	F3165	1: 10000

Supplementary Table 2. Primer sequences used for ChIP-qPCR (a) and q-RTPCR (b)

a. List of primer sequences for ChIP-qPCR

ChIP_JMJD3	Sense Primer	Anti-sense Primer
Tfeb	GCATGTTCTACTGTGTGAGG	CCACACTCCCCAAAGGTCA
Pgc1α	CACCATTGTTAGTTAGCAGC	ATGTTCTCGGGGAAAGTTTG
Ulk1	GAACCCCATTCTCAGTAAGG	AAGGCGGCCATCAACACTAA
Map1lc3a	AGACAGAAAGCTCCAAAGGC	TAGCACGGGTGTTCTGTGTA
Atg3	TAGGCTACATACGGTAACTTG	AGATCGAACAGCCTACTCTG
Atg7	TTGAGCCAAACCCAAACAATG	AAGACAGATGTTCCAGGCAG
Bnip1	GGCTCTCTAGCAGACAACAA	GACATCTCACCCAGTAATGC
Fgf21	TTGGAAATGGAGGGAACTTTG	AAGTTGTTGGTCCACTGTCC
Atgl	TGTAAGCTACTGTTCTTGAGG	CTAACATGGTATGGCACGTG
Hsl	AGAGGGTGATGCTGTCTTAC	CTAAATCGGTATTGTAGCCAC
**ChIP_H3K27me3**	**Sense Primer**	**Anti-sense Primer**
Tfeb	CTTCTCACTATTCAGCTTCC	TCATTCAGGTACTGGGCAG
Pgc1α	ATGTTCACACCTTCTCGGC	ACTGGCTGTGGGTTTTCAG
Ulk1	CTGGGAAAGCCATGTGGAT	TGAGGGCAAACATACCTTAC
Map1lc3a	ATGATTAATGTGGGAGGTCC	GCTGTATACTGGCTTGCTC
Atg3	GCTGACTTTCCTCCCTACT	ATGAGGTGAGAAAGCTAAGG
Atg7	CGCTTTACTCCCAGTCTGA	AAGGCTCATTGGCACAAGG
Bnip1	GTGAGGTGGTTGGTTAGAG	GGAACTCCAGCTGCATATC
Fgf21	CTTGAGTGTTAGGGCTCCA	GTTCCTGTACTTGTAGTTGG
Atgl	GTAGGCATTTATTTGCTCGC	AGACAGAGATGCGTGCGAT
Hsl	GTGAGTTCAAGGCCAGTCT	ATTGAGGATAGACCCCAGG

b. List of primer sequences for q-RTPCR

mouse	Sense Primer	Anti-sense Primer
36b4	CGACATCACAGAGCAGGC	CACCGAGGCAACAGTTGG
Tfeb	CTCAGTTTCTCCTTGTGC	GTATTCATCATCTTCGTAGTCT
Ulk1	ACCATTGTCTACCAGTGT	AGTGTCTTGTTCTTCTCATAA
Map1lc3a	AGAAGGATGAAGACGGATT	TAGTTTAGGAGCCAGGAC
Atg3	TCACAACACAGGTATTACAG	CTTCCTCGTCTTCTTCATC
Atg7	CAGAAGAAGTTGAACGAGTA	CAGAGTCACCATTGTAGTAAT
Bnip1	AACAGAGCGAGGAGGCTAT	CTTTGTGATGAGCTTCCGG
Fgf21	AACAACACCATCCCTCCCA	GGGCTTCTGCCTCATTTTC
Atgl	AAGAGCAGACGGGTAGCAT	AGTTCCACCTGCTCAGACA
Hsl	GAACCCCTTCATGTCTCCT	ATCTAGCATGGGGTCCAGA
Jmjd3	AAGATCGCTTACCAAGGCC	TCTCACTTGTAACGAACAGG
Jmjd3 (pre-mRNA)	AGTGCTCATACTAACCAAGG	TCAGCCTCAGAAGTGTGTC
Ezh2	GCTAAGAGGGCTATCCAGA	CAAGGGATTTCCATTTCTCG
Utx	GGATCTGATGCAAGTCTATG	TGCAGAACTGGGTTACTTTC
Klb	CGAGCCCATTGTTACCTTGT	CTCCAAAGGTCTGGAAGCAG
Fgfr1	CTGAAGGAGGGTCATCGAAT	GTCCAGGTCTTCCACCAACT
Fgfr4	GACCAAACCAGCACCGTGGCTGTGAAGATG	GTTTCCCTTGGCGGCACATTCCACAATCAC
Pgc1α	TTCGAGCTGTACTTTTGTGG	TACCTGCGCAAGCTTCTCT
**human**	**Sense Primer**	**Anti-sense Primer**
36B4	TTGGCTACCCAACTGTTGCA	CACAAAGGCAGATGGATCAGC
TFEB	CTGCAAAAGTCCAGGGAGCT	ATGTTCATGCCGGACGGG
ATG7	TGCATCAAGAAACCCAAGCTG	CTGCTCTGGCGATGGAGAG
ATGL	GTGGATGAAGGAGCAGACGG	GGCAGTGCCTCTCTGTAGG
βKL	ACATTTACATCACCGCCAG	AGATTTCTCTTCAGCCAGTT
JMJD3	GAAAATCGCTTACCAGGGC	GGCTGCCATTCTCACTTGT
ULK1	TTTGGCCGGTCTTTCAGCA	GCTTCTCCTGCAGGCTCTC
FGFR1	ACATTCACCACATCGACTAC	GAGTGAAGATCTCCCACAG
